# A review of machine learning in scanpath analysis for passive gaze-based interaction

**DOI:** 10.3389/frai.2024.1391745

**Published:** 2024-06-05

**Authors:** Abdulrahman Mohamed Selim, Michael Barz, Omair Shahzad Bhatti, Hasan Md Tusfiqur Alam, Daniel Sonntag

**Affiliations:** ^1^German Research Center for Artificial Intelligence (DFKI), Interactive Machine Learning Department, Saarbrücken, Germany; ^2^Applied Artificial Intelligence, University of Oldenburg, Oldenburg, Germany

**Keywords:** machine learning, eye tracking, scanpath, passive gaze-based interaction, literature review

## Abstract

The scanpath is an important concept in eye tracking. It refers to a person's eye movements over a period of time, commonly represented as a series of alternating fixations and saccades. Machine learning has been increasingly used for the automatic interpretation of scanpaths over the past few years, particularly in research on passive gaze-based interaction, i.e., interfaces that implicitly observe and interpret human eye movements, with the goal of improving the interaction. This literature review investigates research on machine learning applications in scanpath analysis for passive gaze-based interaction between 2012 and 2022, starting from 2,425 publications and focussing on 77 publications. We provide insights on research domains and common learning tasks in passive gaze-based interaction and present common machine learning practices from data collection and preparation to model selection and evaluation. We discuss commonly followed practices and identify gaps and challenges, especially concerning emerging machine learning topics, to guide future research in the field.

## 1 Introduction

**Figure 1:**
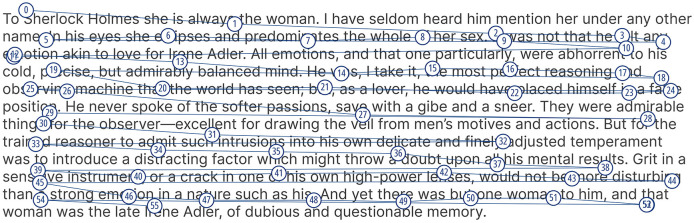
An example of a real scanpath visualization during a reading task.

Eye tracking is a technology that records eye movements and gaze locations over time (Carter and Luke, 2020), and has seen increased usage in research over recent years. The scanpath is an important concept in eye tracking, and it refers to the trace of a user's eye movements across space over a period of time (Holmqvist et al., 2011). Scanpaths are closely associated with two eye tracking terms: fixations and saccades. Fixations describe the state when the eyes remain relatively still for a time period lasting between a few tens of milliseconds up to a few seconds, while saccades are the rapid eye movements from one fixation to another (Holmqvist et al., 2011). The combination of both fixations and saccades produces a scanpath. Figure 1 shows a visual encoding of a scanpath where the numbered circles represent fixations, the lines connecting them represent saccades, and both are superimposed on top of a text stimulus. Scanpaths are often regarded as one of the most commonly used methods for analyzing and representing human eye movements (Blascheck et al., 2017; Li et al., 2021).

**Figure 2:**
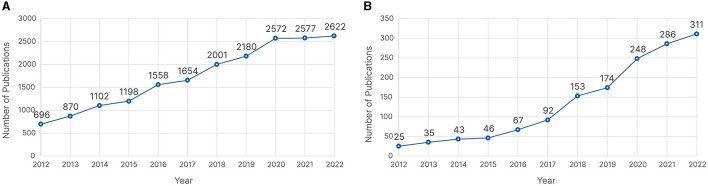
Number of publications from 2012 to 2022 retrieved from Dimension AI on 27 November 2023 for **(A)** eye tracking as whole and **(B)** eye tracking alongside machine learning.

The latest Artificial Intelligence (AI) Index Annual Report revealed that AI research has more than doubled since 2010 (Maslej et al., 2023). This prompted us to investigate whether this trend can also be found in eye tracking research. A query to the Dimensions AI database^1^ for eye tracking publications^2^ from 2012 to 2022 showed an almost fourfold increase in the number of publications, as shown in Figure 2A. This growth has occurred despite a slowdown due to the COVID-19 pandemic. By further refining our search query to include some common machine learning (ML) keywords,^3^ we observed a rapid increase in publications, with more than a tenfold increase from 2012 to 2022, as shown in Figure 2B. This shows that the eye tracking community has been rapidly adopting ML algorithms in their research, aligning with the statement of Maslej et al. (2023). These findings encouraged us to conduct a literature review where we focus on a specific topic within eye tracking research to provide an overview of how ML has been used. Because of the importance of scanpaths and because automated scanpath analysis has been under investigation since the late nineties (Brandt and Stark, 1997), we decided to focus on scanpaths for passive gaze-based interaction, Passive gaze-based applications use eye tracking as a supporting modality to monitor and understand a user's behavior without the user actively interacting with the system using their gaze (Qvarfordt, 2017; Duchowski, 2018).

This paper provides a practical overview of how ML has been used for scanpath analysis in passive gaze-based applications. Our contribution lies in reporting and summarizing findings from 77 publications between 2012 and 2022. We begin by examining the different research domains and passive gaze-based learning tasks to see the current trends in the field. Afterwards, we structure our ML findings in the order of a general ML workflow based on those from Amershi et al. (2019) and Souza et al. (2022). An ML workflow is a high-level overview of the different steps needed for an ML project. It consists of three main steps: the data curation, where a dataset is acquired; the learning data preparation, where the dataset is transformed into a proper format ready to be processed by the ML algorithm; and the learning process, where the ML algorithm is trained on the dataset, and is evaluated afterwards. We provide a comprehensive overview of how ML has been used in scanpath analysis for passive gaze-based interaction to highlight research gaps that could benefit from further investigation; we do so by providing answers to these questions:

Q1. What research domains have used machine learning to analyze scanpaths, and what passive gaze-based learning tasks have they focused on?
Q2. Which research domains and passive gaze-based learning tasks have yet to use machine learning in scanpath processing?
Q3. What are the commonly followed machine learning practices, in line with a general machine learning workflow, that have been used for scanpath analysis in passive gaze-based applications?
Q4. Which machine learning topics have yet to be investigated for scanpath analysis in passive gaze-based interaction, and what benefits could they provide?

## 2 Review methodology

**Figure 3:**
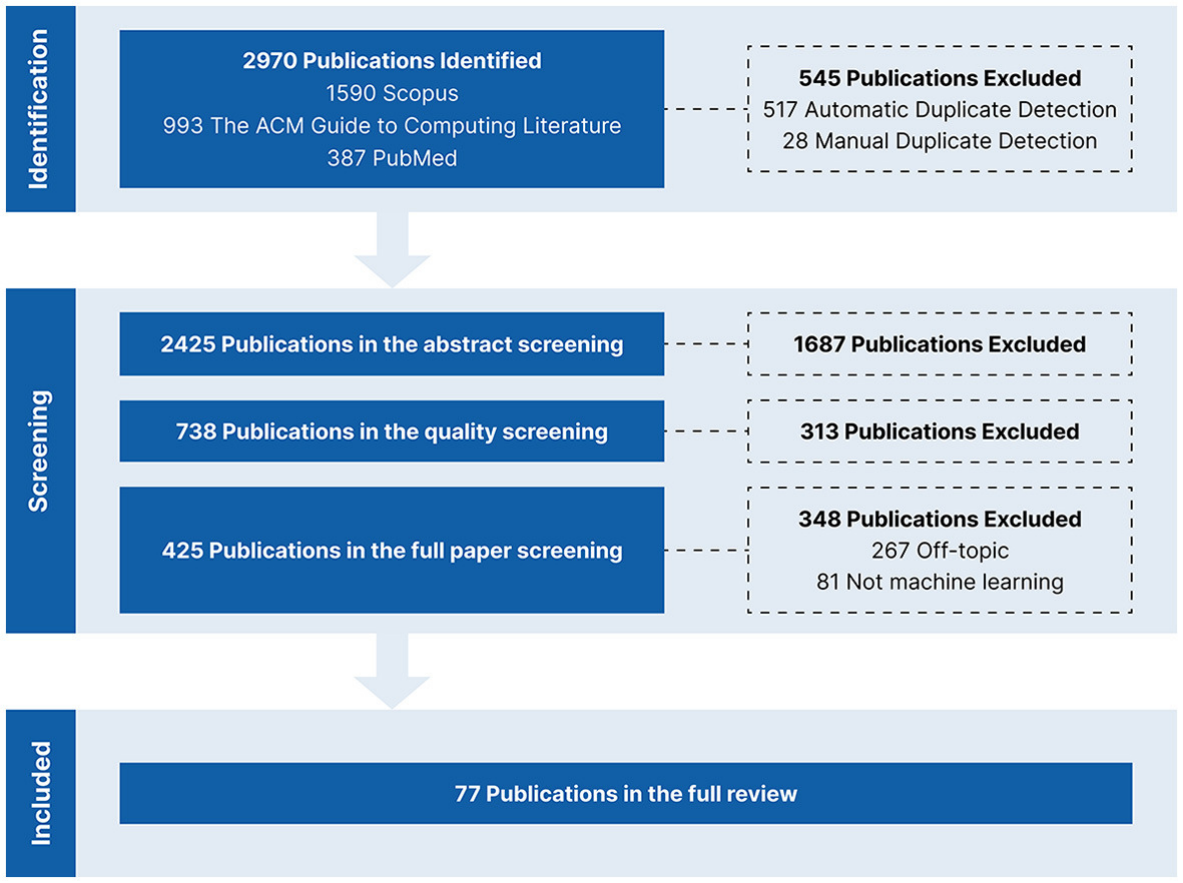
PRISMA flow diagram.

A literature review mainly consists of four main phases: the identification phase, where we retrieve publications from databases using keywords; the screening phase, where we classify the retrieved publications as either relevant or irrelevant to our topic; the data extraction phase, where we extract the information and insights that we want to report from the relevant publications; and the reporting phase where we report our findings. We followed the PRISMA^4^ framework (Page et al., 2021) throughout the review to make sure we did not overlook any step in our reporting. Figure 3 shows our PRISMA flow diagram to summarize and provide an overview of the different phases in our review.

### 2.1 Identification

We searched for English research articles on eye tracking and scanpaths published between 2012 and 2022 in three databases: Scopus,^5^ The ACM Guide to Computing Literature,^6^ and PubMed.^7^ This is in line with the AMSTAR^8^ guidelines of querying at least two databases for a literature review (Shea et al., 2017). Our search query consisted of two terms connected via an AND operator, and each term consisted of multiple keywords connected via an OR operator as follows: (“Eye Tracking” **OR** “Eye-Tracking” **OR** “Eyetracking” **OR** “Eye Movement”) **AND** (“Scan Path*” **OR** “Scanpath*” **OR** “Visual Scanning” **OR** “Gaze Pattern”). We defined the query based on a preliminary search of related publications. We included the word stem for the most common spellings of Scanpath and Eye Tracking. Adding an asterisk * allows search engines, with regular expression support, to return results containing the singular or plural of a word and corresponding compound words. In addition, to make sure we did not overlook any publication, we included the keywords Gaze Pattern and Eye Movement. We decided not to include any ML keywords in the query because we did not want to overlook or miss relevant publications; ML is very diverse, and we might have gotten limited results if we had only focused on certain keywords. The databases retrieved publications with at least one of the keywords in their title or abstract. As shown in Figure 3, we retrieved 2,970 publications from all three databases. We used the Zotero^9^ reference management tool to automatically detect duplicates, which we manually double-checked. We discarded 545 duplicates: 517 were identified by Zotero, and 28 by manual inspection.

### 2.2 Screening

We started the screening phase with 2,425 publications. We conducted the initial screening step based on the abstracts. We discarded any review papers, workshop papers, demo papers, extended abstracts, book chapters, and any publication that was clearly out of scope. We ended the initial screening by discarding 1,687 publications and retaining 738 publications for further analysis.

Afterwards, we conducted an intermediate screening step based on the venue quality.^10^ For journals, we only retained journals with an impact factor of three or higher. For conferences, we only retained A-ranked conferences. In addition, to ensure that we did not discard any specialized venues regardless of their rank or impact factor, we retained any venue, whether journal or conference, with five or more publications. We conducted the quality-based intermediate screening to ensure that we focused on publications from top venues. This might have led to discarding some relevant papers, but we believe this step was crucial for quality assurance. We ended the intermediate screening by discarding 313 publications and retaining 425 publications.

For the final screening step, we screened the full papers to make sure they were within our scope and that they used ML to process scanpaths. We discarded 267 publications that were not passive gaze-based applications or for scanpath processing, and 81 publications that did not use ML for scanpath processing. We ended the screening phase with 77 publications for information extraction.

### 2.3 Information extraction

We started the information extraction phase with 77 publications, which included 48 journal publications and 29 conference publications. The top three venues with respect to the number of remaining publications were The ACM Symposium on Eye Tracking Research and Applications, (ETRA)^11^ The ACM International Conference on Multimodal Interaction (ICMI),^12^ and Vision Research Journal.^13^ Moving forward, we present the different insights we extracted from the publications in the order of the ML workflow shown in Figure 6. However, we first discuss the research domains and learning tasks across the 77 publications. This step precedes the ML workflow but should help identify the current research state and possible challenges and gaps. We attached an Excel sheet as Supplementary material to this review, which includes all the information extraction phase details. It includes the publications' metadata, research domain categorization, learning tasks, used ML algorithms, and extracted scanpath features. In addition, it lists all experiments and corresponding results per publication.

## 3 Domains and tasks

Across the 77 publications included in this review, we identified four main research domains and ten main passive gaze-based learning tasks, with an additional domain and task labeled as **Other** for those with fewer than three publications each. We categorized publications based on the ML task of interest for which the scanpaths were processed, which led to some publications falling into multiple domains and tasks. We begin this section with an overview of the research domains, followed by the learning tasks.

### 3.1 Research domains

**Figure 4:**
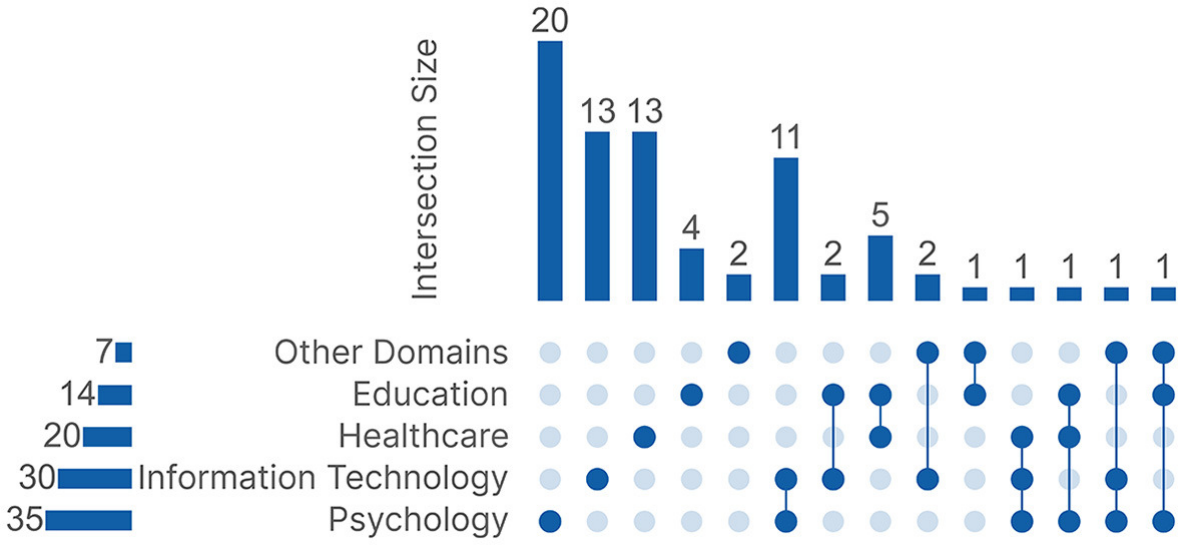
The UpSet plot between the five research domains.

Research domains present a high-level perspective on the focus of studies on ML for scanpath processing in passive gaze-based applications. We found that the publications primarily fell under five research domains: *Education, Healthcare, Psychology, Information Technology*, and *Other Domains*, which includes domains with fewer than three publications each. Each publication was categorized based on what it wanted to infer from the ML results. Some publications focused on multiple tasks, which led them to be categorized under multiple domains. Figure 4 shows an UpSet Plot (Lex et al., 2014) to visualize the different connections between all five research domains. Of the 77 publications, 52 were categorized under a single domain, and 25 were categorized under multiple domains.

#### 3.1.1 Education (*n* = 14 publications)

*Education* focuses on publications directed toward teaching and learning processes. In addition to designing and evaluating educational material, theories, and technologies. For example, how dental students from different semesters differ in their visual behavior while viewing medical scans (Castner et al., 2018) or predicting learning behavior while doing complex tasks (Giannakos et al., 2019).

#### 3.1.2 Healthcare (*n* = 20 publications)

*Healthcare* focuses on diagnosing various health conditions, such as vision loss (Crabb et al., 2014) and different psychiatric, learning, neurodevelopmental, and mood disorders (Atyabi et al., 2023). In addition to any task related to healthcare professionals, such as comparing their visual behavior (Castner et al., 2018), or monitoring their attention (Khosravan et al., 2019).

#### 3.1.3 Psychology (*n* = 35 publications)

*Psychology* holds a wide range of publications aimed at understanding and predicting human behavior and cognitive processes. This includes exploring perceptual and behavioral differences across different groups (Abdi Sargezeh et al., 2019), monitoring stress levels (Kim et al., 2022) and cognitive load (Ktistakis et al., 2022), assessing reading behaviors (Kelton et al., 2019), understanding emotion perception and attention patterns (Kanan et al., 2015), and predicting user tasks and decisions in various contexts (Coutrot et al., 2018). The domains *Psychology* and *Healthcare* are closely related. We assigned a publication to *Healthcare* if it focused on diagnosis only. If the underlying visual behavior was investigated as well, such as in Hayes and Henderson (2018), we assigned both domains.

#### 3.1.4 Information Technology (*n* = 30 publications)

*Information Technology* has two main types of publications: those that focus on the technical implementation and the methodology more than the actual task, e.g., Li et al. (2021), and those that focus on technology tasks such as assessing password strength (Abdrabou et al., 2021), user experience (UX) evaluation (Moon et al., 2021), adaptive visualizations (Fu and Steichen, 2022), and contributing to fields like affective computing and cognitive modeling (Alghofaili et al., 2019).

#### 3.1.5 Other Domains (*n* = 7 publications)

*Other Domains* holds various publications which could not be clearly assigned to only one of the other four domains. This includes publications related to driving (Lethaus et al., 2013), aviation (Peysakhovich et al., 2022), maritime (Li et al., 2022), marketing and product design (Moacdieh and Sarter, 2017), and linguistics (Reich et al., 2022).

### 3.2 Learning tasks

**Figure 5:**
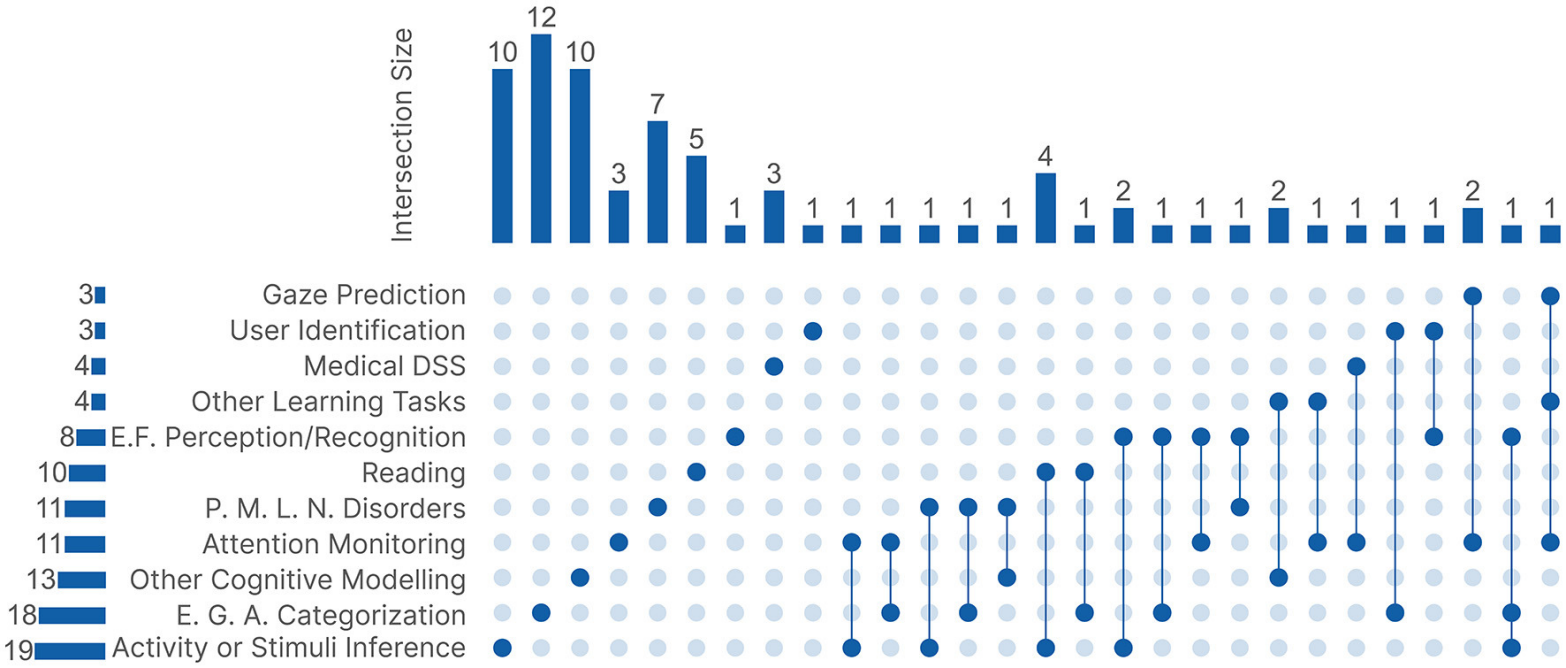
The UpSet plot between the 11 learning tasks. Medical DSS, Medical Decision Support System; E.F., emotion or face; P.M.L.N., psychiatric, mood, learning, or neurodevelopmental; E.G.A, Experience, Gender, or Age Categorization.

Learning tasks offer a more detailed perspective on the research focus across the 77 publications. Similar to the research domains, some publications were categorized under multiple learning tasks. This was mainly because of publications that developed novel processing methods and tested them on different tasks. We had ten main task groups alongside an extra group **Other Tasks**, which includes tasks with fewer than three publications each. Figure 5 shows an UpSet Plot to visualize the connections between all 11 learning tasks. Similar to the research domains, out of the 77 publications, 52 were categorized under only one learning task.

#### 3.2.1 Activity, or Stimuli Inference (*n* = 19 publications)

**Activity, or Stimuli Inference** holds publications that focus on predicting the activity or stimulus based on the assumption that different stimuli and activities produce different scanpath data. For activity prediction, the activities were often diverse, including viewing natural images, web surfing, or watching a video (Greene et al., 2012; Kanan et al., 2014; Haass et al., 2016; Martinez et al., 2017; Coutrot et al., 2018; Hild et al., 2018; Srivastava et al., 2018; Kucharský et al., 2020; Lan et al., 2020). However, some publications focused on specific tasks such as driving cars (Lethaus et al., 2013), piloting plans (Peysakhovich et al., 2022), or reading text (Biedert et al., 2012; Kelton et al., 2019). For stimuli prediction, publications used different videos, images, or text types to study the visual behavior unique to each stimulus (Greene et al., 2012; Lanatà et al., 2013; Kanan et al., 2014; Coutrot et al., 2016, 2018; Fuhl et al., 2019; Necka et al., 2019; Lan et al., 2020; Wang et al., 2020; Li et al., 2021).

#### 3.2.2 Experience, Gender, or Age Categorization (*n* = 18 publications)

**Experience, Gender, or Age Categorization** holds publications that focus on the assumption that certain groups of people have unique visual behavior characteristics. This was used to differentiate between different genders (Coutrot et al., 2016; Galdi et al., 2016; Abdi Sargezeh et al., 2019; Atyabi et al., 2023), and age groups (Glady et al., 2013; Galdi et al., 2016; Chaby et al., 2017; French et al., 2017; Atyabi et al., 2023). Other publications focused on identifying characteristic visual behavior associated with different levels of experience within certain professions such as dentists (Castner et al., 2018, 2020, 2022), radiographers and radiologists (Gandomkar et al., 2017, 2018; Li et al., 2019), pilots (Lounis et al., 2021), and students (Pejić et al., 2021). While others focused on different levels of experience, but within a certain task such as history learning (Sáiz-Manzanares et al., 2021), crossword puzzles (Sáiz Manzanares et al., 2020), and coding comprehension (Harada and Nakayama, 2021).

#### 3.2.3 Attention Monitoring (*n* = 11 publications)

**Attention Monitoring** holds publications that focus on monitoring the visual attention behavior over the course of a specific activity or a period of time (Jiang et al., 2016; Shi et al., 2017; Xu et al., 2018; Abdelrahman et al., 2019; Khosravan et al., 2019; Lounis et al., 2021; Xia et al., 2021; Peysakhovich et al., 2022). This was sometimes related to other tasks, such as reaction or response time prediction (Moacdieh and Sarter, 2017; Li et al., 2022), or modeling a participant's conversation engagement (Ishii et al., 2013).

#### 3.2.4 Emotion, or Face Perception/Recognition (*n* = 8 publications)

**Emotion, or Face Perception/Recognition** holds publications that focus on the visual behavior associated with viewing different faces or emotion-inducing stimuli to predict the recognized face or the perceived emotion. These publications explicitly used stimuli depicting different emotions, such as anger and happiness (Lanatà et al., 2013; Kanan et al., 2015; Chaby et al., 2017; Shi et al., 2017), or different people's faces (Coutrot et al., 2016; Chaby et al., 2017; Chuk et al., 2017a; Król and Król, 2019; Necka et al., 2019). This task group focused explicitly on those types of stimuli, which meant that if a publication focused, for example, on different activities to induce certain emotions, it was not included in this task group.

#### 3.2.5 Reading (*n* = 10 publications)

**Reading** holds publications that focus on aspects related to reading behavior. It is built on the assumption that reading could be a unique indication toward understanding a participant's or a text's characteristics. This includes predicting text difficulty (Wang et al., 2020; Reich et al., 2022), text relevance to a trigger question (Bhattacharya et al., 2020a), the type of text document (Lan et al., 2020), reading comprehension (Reich et al., 2022; Southwell et al., 2022), whether the reader is a native speaker of the text language (Reich et al., 2022), and whether the participant is reading or skimming the text (Biedert et al., 2012; Kelton et al., 2019). In addition to specialized use cases such as understanding visual behavior indicative of correct and incorrect responses to a reading task (Nakayama and Hayashi, 2014), evaluating computer science skills (Harada and Nakayama, 2021), and evaluating sarcasm understandability (Mishra et al., 2016).

#### 3.2.6 Other Cognitive Modeling (*n* = 13 publications)

**Other Cognitive Modeling** holds publications that focus on cognitive modeling tasks. However, the previous learning tasks could fully or partially be considered forms of cognitive modeling. This task group does not include any of the learning tasks from any of the previous groups. This group holds tasks such as predicting confidence (Smith et al., 2018), confusion (Sims and Conati, 2020), stress (Kim et al., 2022), cognitive workload (Ktistakis et al., 2022), navigation aid requirement (Alghofaili et al., 2019), and cognitive processing (Raptis et al., 2017; Roy et al., 2020). In addition, it holds publications that focus on understanding the visual behavior indicative of success or failure (Giannakos et al., 2019; Appel et al., 2022; Fu and Steichen, 2022), modeling how different disorders affect the viewing behavior of natural images (Hayes and Henderson, 2018), evaluating gaze behavior associated with weak and strong password creation (Abdrabou et al., 2021), and perception of product design (Moon et al., 2021).

#### 3.2.7 Psychiatric, Mood, Learning, or Neurodevelopmental Disorders (*n* = 11)

**Psychiatric, Mood, Learning, or Neurodevelopmental Disorders** holds publications that focus on different tasks related to multiple disorders such as Autism Spectrum Disorder (ASD) (Hayes and Henderson, 2018; Król and Król, 2019; Liaqat et al., 2021; Li et al., 2021; Kanhirakadavath and Chandran, 2022; Varma et al., 2022; Atyabi et al., 2023), schizophrenia (Benson et al., 2012; Nikolaides et al., 2016), bipolar disorder (Chung et al., 2018), depression (Chung et al., 2018; Zhang et al., 2022), Attention Deficit Disorder (ADD) (Hayes and Henderson, 2018), and dyslexia (Hayes and Henderson, 2018).

#### 3.2.8 Medical Decision Support System (*n* = 4 publications)

**Medical Decision Support System** holds publications that focus only on medical tasks, such as diagnosing vision loss (Crabb et al., 2014; David et al., 2019; Krishnan et al., 2021) and understanding visual attention on medical images (Khosravan et al., 2019). There is an overlap between this task group and the previous task group, i.e., **Psychiatric, Mood, Learning, or Neurodevelopmental Disorders**. However, due to the nature of the publications in the previous task group, we did not want to add them underneath **Medical Decision Support Systems**. So, we created a distinction between both groups.

#### 3.2.9 User Identification (*n* = 3 publications)

**User Identification** holds publications that focus on collecting data from multiple participants to try and identify each participant based on their unique visual behavior (Kanan et al., 2015; George and Routray, 2016; Pejić et al., 2021).

#### 3.2.10 Gaze Prediction (*n* = 3 publications)

**Gaze Prediction** holds publications that focus on predicting future gaze behavior based on previous gaze behavior depicted in scanpath data (Jiang et al., 2016; Xu et al., 2018; Xia et al., 2021).

#### 3.2.11 Other Tasks (*n* = 4 publications)

*Other Tasks* holds various publications that could not be clearly assigned to only one of the other 11 learning tasks. This includes security tasks for evaluating password strength (Abdrabou et al., 2021), evaluating product design and user experience (Moacdieh and Sarter, 2017; Moon et al., 2021), and visual search (Xu et al., 2018).

## 4 Machine learning and scanpaths

**Figure 6:**
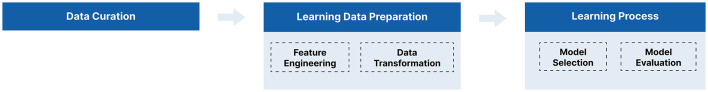
A general machine learning workflow.

Our goal in this review is to present how ML is used to process scanpaths for passive gaze-based applications. The previous section explored the different research domains and passive gaze-based learning tasks. This section focuses on the ML process, which is organized according to an ML workflow. Figure 6 shows the workflow that we followed, which is based on the workflows from Amershi et al. (2019) and Souza et al. (2022). This workflow is divided into three main parts: Data Curation, Learning Data Preparation, and Learning Process.

In the Data Curation, we outline the specifications for both the apparatus and the participants involved in the data collection studies. Additionally, we report on the use of external datasets by some publications as an alternative to conducting user studies. In the Learning Data Preparation, we discuss the various scanpath representation formats and features, and the different strategies for partitioning the data into training and testing subsets for ML algorithms. In the Learning Process, we examine the different ML algorithms, with a more detailed focus on neural networks, and evaluation metrics reported across the various publications. We present our findings throughout each step and draw connections, when appropriate, to the different research domains and ML algorithms. This should provide a more tailored experience across the full topic of interest and highlight the standard practices followed in the field.

### 4.1 Data Curation

This section focuses on the practical steps required to prepare a properly labeled and ready-to-use dataset. We report the specifications of the used eye trackers and information regarding participant selection to help guide an informed decision. In addition, we report the external datasets used by some publications.

#### 4.1.1 Apparatus

An important step in eye tracking studies is to decide on the eye tracker frequency. We could not find consistent reporting across all 77 publications because some studies reported the frequency of the eye tracker, others reported the downsampling frequency, and a few reported the eye tracker model without detailing any frequencies. For studies that reported a downsampling frequency, it was used instead of the data collection frequency. For those that did not report a frequency, the default frequency of the device found online was documented. If a publication used multiple datasets or conducted separate user studies, we treated each one separately. However, if a publication conducted a single user study and later examined different use cases using the same dataset, it was recorded only once.

**Figure 7:**
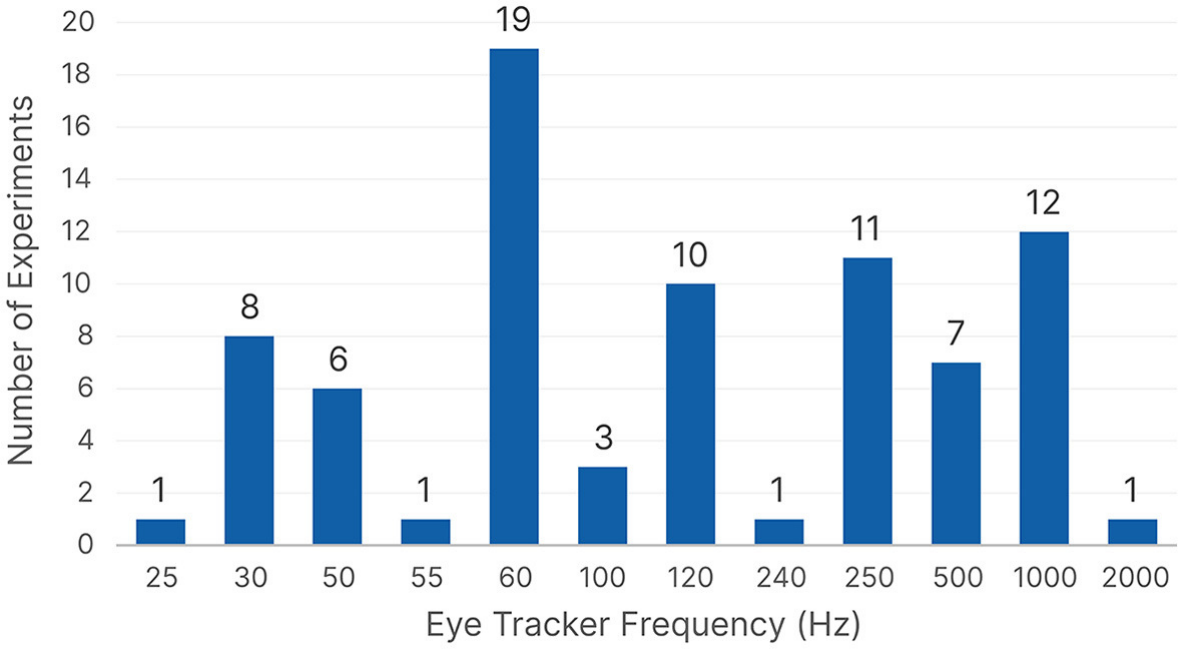
The eye tracker frequencies count based on the reported user studies.

Figure 7 shows the distribution of the eye tracker frequencies. We can see that 60 Hz is the most frequently used eye tracker frequency, followed by 1,000 Hz. However, most studies that reported a downsampling frequency used a 1,000 Hz eye tracker, which was then downsampled to either 500 Hz (Benson et al., 2012; Mishra et al., 2016; Necka et al., 2019; Kucharský et al., 2020) or 250 Hz (Coutrot et al., 2016, 2018; George and Routray, 2016). Upon further investigation, we found that 60 Hz was the most common frequency across all five domains except for *Psychology*, which had 1,000 Hz as the most popular choice. When we considered the type of ML algorithm used afterwards in the processing, we found that 60 Hz was still the most common frequency for traditional ML, but 120 Hz was the most common choice for neural networks. We can see that across almost all domains and ML algorithms, 60 Hz is the most common choice, followed by 1,000, 250, and 120 Hz, respectively.

#### 4.1.2 Participant demographics and group distribution

**Figure 8:**
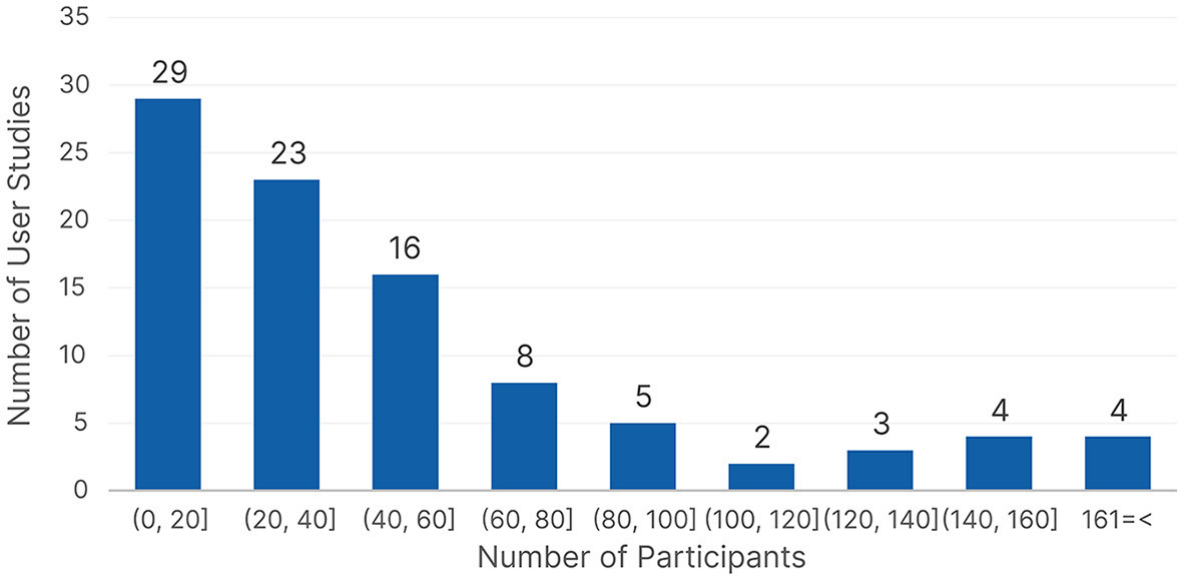
The number of participants count based on the reported user studies.

Another important aspect of user studies is to determine the number of participants. We found a total of 94 reported datasets across the different publications because a few publications conducted multiple user studies or used more than one dataset. Figure 8 shows the number of participants reported across the different datasets, we had 57 unique dataset sizes, so we grouped them to provide better insights. More than 50% of the reported datasets used data from up to 40 participants. We found that using 24 participants (*n* = 8), followed by eight participants (*n* = 7) were the most popular single values. Only three publications (Benson et al., 2012; Coutrot et al., 2016; Appel et al., 2022) used more than 200 participants; all three publications used existing datasets and did not conduct their own user studies. Upon further investigation, we found that *Information Technology, Psychology*, and *Education* preferred using data with up to 40 participants, but *Healthcare* preferred larger datasets with 61–80 participants. The insights did not change drastically when looking at the data with respect to the type of ML algorithm, with collecting data from up to 40 participants being the most frequent strategy for both traditional ML and neural network algorithms. We can see that there is a tendency to collect data from up to 40 participants, with a slight preference for around 20 participants throughout the different domains and ML algorithms.

Out of the 94 reported datasets, only 44 reported the number of females and males. Fifteen datasets were balanced, with female participants making between 40 and 55% of the data. Only eight datasets were skewed toward having more female participants, with female participants making up between 60 and 80% of the data. While 21 datasets had more male participants, with female participants making up <40% of the data. We had 27 datasets with distinct control and target groups. For any diagnosis, the healthy participants were the control group; for any experience categorization, the experienced professionals were the control group; and for age categorization, older participants were the control group. Sixteen datasets had an almost balanced split, with the target group making between 40 and 55% of the data, with seven datasets having a perfect 50-50 split. The target group across the remaining 11 datasets made between 56 and 78% of the data.

Reporting the demographic information of the dataset is important because it could lead to future contributions. For example, if a study wants to focus on data from only one gender or for participants belonging to a certain age group and so on. In addition, diverse datasets concerning gender and age have a better chance of providing insights that could lead to better generalizability. We could not provide similar insights toward the number of stimuli or participants' ages, because they were not consistently reported. Some publications reported the number of trials, some reported the length of the recording, and others reported the number of collected gaze points. There was an inconsistency in the reporting for us to extract meaningful insights, which could also come back to the range of learning tasks the publications covered.

#### 4.1.3 External datasets

Eighteen publications opted to use already available datasets for a total of 23 different reported datasets. Only three datasets were used by two different publications: the OSIE dataset (Xu et al., 2014), the MIT dataset (Judd et al., 2009), and the dataset from Coutrot and Guyader (2014). While the publications for the SedentaryActivity dataset (Srivastava et al., 2018) and the dataset from Greene et al. (2012) are both covered within this review. Table 1 lists all 23 datasets alongside the learning tasks that they were used for. We wanted to report these datasets because they might be available online or via correspondence with their respective authors. This could help researchers save the tedious time, effort, and expenses required for collecting a dataset and conducting a user study.

**Table 1:** Table listing the 23 reported external datasets, alongside the learning tasks they were used for and by which publication.

**Dataset**	**Learning task**
The OSIE dataset (Xu et al., 2014)	Attention Modeling & Gaze Prediction (Jiang et al., 2016; Xia et al., 2021)
The MIT dataset (Judd et al., 2009)	Attention Modeling & Gaze Prediction (Jiang et al., 2016; Xia et al., 2021)
	Stimuli Inference (Li et al., 2021)
The ETRA 2019 challenge^a^ (Otero-Millan et al., 2008; McCamy et al., 2014)	Stimuli Inference (Fuhl et al., 2019)
The JapaneseDocument dataset (Kunze et al., 2013)	Stimuli Inference (Lan et al., 2020)
Dataset from Coutrot and Guyader (2014)	Stimuli Inference (Coutrot et al., 2018)
	Training the OBF Framework (Li et al., 2021)
Dataset from Gitman et al. (2014)	Training the OBF Framework (Li et al., 2021)
Dataset from Coutrot and Guyader (2015)	Training the OBF Framework (Li et al., 2021)
The SedentaryActivity dataset (Srivastava et al., 2018)	Activity Inference (Lan et al., 2020)
Dataset from Koehler et al. (2014)	Activity Inference (Coutrot et al., 2018)
Dataset from Laurence et al. (2018)	Activity Inference (Kucharský et al., 2020)
Dataset from Trutescu and Raijmakers (2019)	Activity Inference (Kucharský et al., 2020)
Dataset from Greene et al. (2012)	Activity and Stimuli Inference (Kanan et al., 2014)
The TüEyeQ dataset (Kasneci et al., 2021)	Cognitive Modeling (Appel et al., 2022)
Dataset from Lallé et al. (2016)	Cognitive Modeling (Sims and Conati, 2020)
Dataset from Chuk et al. (2014)	Face Perception/Recognition (Chuk et al., 2017a)
Dataset from Chuk et al. (2017b)	Face Perception/Recognition (Chuk et al., 2017a)
Dataset from Galdi et al. (2013)	Gender and Age Categorization (Galdi et al., 2016)
The EMIP dataset (Bednarik et al., 2020)	Reading & Experience Categorization (Harada and Nakayama, 2021)
The Stony Brook SAT reading fixation dataset (Ahn et al., 2020)	Reading (Reich et al., 2022)
Dataset from Mills et al. (2021)	Reading (Southwell et al., 2022)
Dataset from Carette et al. (2019)	ASD Diagnosis (Kanhirakadavath and Chandran, 2022)
Dataset from Duan et al. (2019)	ASD Diagnosis (Liaqat et al., 2021)
The BioEye 2015 competition^b^	User Identification (George and Routray, 2016)

a https://etra.acm.org/2019/challenge.html.

b https://bioeye.cs.txstate.edu/.

### 4.2 Learning data preparation

In the learning data preparation, we focus on preparing the dataset as a suitable input to the ML algorithm. This section focuses on the different scanpath representation formats and the different methods for splitting a dataset into training and testing subsets. This is part of formulating the standard ML practices for scanpath processing.

#### 4.2.1 Scanpath representation formats

We found that there were six main formats for representing scanpaths as suitable inputs to an ML algorithm: Visual Encoding (*n* = 10 Publications), String Representation (*n* = 12 Publications), Time Series (*n* = 9 Publications), Graph Representation (*n* = 4 Publications), Feature Engineering (*n* = 64 Publications), and Hidden Markov Modeling (*n* = 6 Publications). **Visual Encoding** is when a scanpath, i.e., the combination of fixations and saccades, is projected on top of either a stimulus, as shown in Figure 1, or an empty space; this is the format most commonly associated with scanpaths. **String Representation** is the name we gave to any sequence of symbols representing a scanpath, which Holmqvist et al. (2011) referred to as symbol sequences; this format includes Area Of Interest (AOI) strings where each symbol represents a fixation or a dwell on an AOI, saccade amplitude and direction-based strings, and fixation duration strings. **Time Series Representation** is when a scanpath is formatted as an ordered series of coordinates, sometimes accompanied by the duration. **Graph Representation** is when the gaze data in a scanpath are clustered or grouped to create nodes and edges representing a graph structure. **Feature Engineering** is a very general term representing when features, e.g., fixation duration, are computed to represent certain aspects of a scanpath. **Hidden Markov Modeling** (HMM) is a type of feature engineering, but because it has been mentioned explicitly by six publications and not as part of feature engineering (Kanan et al., 2014, 2015; Coutrot et al., 2016, 2018; Jiang et al., 2016; Necka et al., 2019), we decided to treat it separately. A HMM is a statistical method used to analyze data that changes over time; the unique feature of HMMs is that they are based on Markov processes, which are memoryless stochastic processes; this means that the probability distribution of the next state depends solely on the current state, not on the sequence of events that led to it (Coutrot et al., 2018).

Feature engineering is the most used format, with 64 publications, but before focusing more on it, we wanted to investigate the other five formats without feature engineering in the picture. We found that the string representation format was the most common representation format for *Education* and tied with time series for *Information Technology*. *Psychology* had the majority of HMM studies, which was tied as the most popular choice, along with time series representation. Lastly, *Healthcare* had visual encoding as the most popular format. When we considered the learning tasks, we found that **Experience, Gender, or Age Categorization** and **Attention Monitoring** were the two tasks with high usage of string representation, while **Activity, or Stimuli Inference** and **Emotion, or Face Perception/Recognition** were the two tasks with high usage of HMM. The remaining tasks were not as frequent in using formats other than feature engineering. Lastly, when we considered the type of ML algorithm, we found that string representation was the most common choice for traditional ML algorithms, while visual encoding was the most common format for neural networks, followed closely by time series data.

##### 4.2.1.1 Feature engineering

We focused on features directly related to scanpaths, fixations, saccades, or AOIs for feature engineering. We excluded any blink features, pupil features, or features computed from additional modalities, such as keyboard keystrokes (Giannakos et al., 2019; Wang et al., 2020), thermal imaging (Abdelrahman et al., 2019), Electroencephalogram (EEG) (Shi et al., 2017; Giannakos et al., 2019; Moon et al., 2021), and physiological monitoring wristbands (Giannakos et al., 2019). Moving forward, when we refer to feature or relevant feature, this is what we mean.

Across the reviewed publications, we found 126 different reported features. Most publications extracted up to 10 features (*n* = 57 Publications) with an average of five features per publication. However, we had two extreme outliers, i.e., Giannakos et al. (2019) extracted 36 features, and Sáiz-Manzanares et al. (2021) extracted 26 features, but both of them used feature selection methods afterwards, especially since Giannakos et al. (2019) had even more features computed from other modalities.

We categorized each feature based on its type into fixation-based, saccade-based, AOI-based, and scanpath. Saccade-based features were the most diverse and numerous (*n* = 63 features). Fixation-based features came in second (*n* = 40 Features), followed by full scanpath features (*n* = 35 Features) and AOI-based features (*n* = 28 Features), respectively. Fixation-based features, e.g., fixation count, and saccade-based features, e.g., saccade duration, require the computation of fixations or saccades, respectively, in order to correctly extract the features. Scanpath features, e.g., scanpath length, require the full scanpath to be constructed in order to compute the feature, and AOI-based features, e.g., fixation count per AOI, take the AOIs into consideration for feature extraction. Some features belong to more than one group, e.g., fixation count per AOI is considered both a fixation-based and an AOI-based feature.

Despite having more saccade-based features, fixation-based features were more commonly used per publication. Average fixation duration was the most commonly used feature (*n* = 24 Publications), followed by total fixation duration (*n* = 16 Publications) and total fixation count (*n* = 16 Publications). Scanpath length (*n* = 13 Publications) was the most common full scanpath feature, followed by total scanpath duration (*n* = 6 Publications). Meanwhile, saccade amplitude (*n* = 7 Publications), total saccade count (*n* = 7 Publications), and total saccade duration (*n* = 7 Publications) were the most commonly used saccade-based features. For AOI-based features, fixation duration per AOI (*n* = 9 Publications) and fixation count per AOI (*n* = 9 Publications) were the most common.

The distribution of the most used features across the research domains and learning tasks showed similar behavior to the overall view, with average fixation duration, total fixation duration, total fixation count, fixation count per AOI, scanpath length, total saccade count, and total saccade duration being the main features used across all five research domains and most of the learning tasks. We found that **Activity, or Stimuli Inference** and **Reading** publications showed very similar tendencies to use the same set of features, which could be due to the fact that multiple publications within the **Activity, or Stimuli Inference** task group used reading as an activity within their user studies. **Experience, Gender, or Age Categorization** publications tended to use a wide range of feature types but were the most frequent users of scanpath and AOI-based features, which are mostly due to using scanpath comparison metrics, where they compare pairs of scanpaths and use the scores as inputs to the ML algorithm. When considering traditional ML and neural networks, we found that traditional ML publications used all feature types. Almost all of the features, i.e., 118 out of 126 features, were for traditional ML algorithms. However, neural networks focused on a less diverse set of features, i.e., 46 out of 126 features.

After the general insights toward the different features, we wanted to highlight three feature groups. The first feature group contains two unique features that were only used by one publication each. Scanpath Spatial Density was only used by Moon et al. (2021), and Saccade Duration Per AOI was only used by Wang et al. (2020). The second feature group contains different algorithms for quantifying the difference between pairs of scanpaths. They were used by 11 publications (Glady et al., 2013; Jiang et al., 2016; French et al., 2017; Shi et al., 2017; Castner et al., 2018; Król and Król, 2019; Li et al., 2019; Necka et al., 2019; Sáiz Manzanares et al., 2020; Liaqat et al., 2021; Appel et al., 2022). Across the publications, we found the following algorithms: Levenshtein Distance (Levenshtein, 1966), Mannan Distance (Mannan et al., 1997), Uniform Distance model, City Block Distance, Euclidian Distance, Hausdorff Distance, Frechett Distance, Vector-Based Comparison (Jarodzka et al., 2010), Dynamic Time Warping (DTW) (Müller, 2007), FastDTW (Salvador and Chan, 2007), Contrast mining (Dong and Bailey, 2012), Needleman-Wunsch Algorithm (Needleman and Wunsch, 1970), in addition to SubsMatch (Kübler et al., 2017) and ScanMatch (Cristino et al., 2010) which both use the Needleman-Wunsch Algorithm. These algorithms were interesting since they were mostly used alone as inputs to the ML algorithms without any additional features. For further reading, the separate sources for each algorithm or systematic reviews focusing on scanpath comparison algorithms, e.g., Anderson et al. (2015); Fahimi and Bruce (2021), could be helpful. The third feature group contains feature extraction methods that were used alone without extracting additional features. Three publications, i.e., Castner et al. (2020); Li et al. (2021); Kanhirakadavath and Chandran (2022), used a Convolutional Neural Network (CNN)-based architecture to extract the features from scanpaths, which were then fed into another ML model for the learning process. Li et al. (2021) presented the Oculomotor Behavior Framework (OBF), which is a framework that makes use of a convolutional, recurrent, and transformer-based architecture to prepare the data and extract features which can then be used to teach other ML models. Hayes and Henderson (2018) used Successor Representation Scanpath Analysis (SRSA) (Hayes et al., 2011, 2015), which uses temporal difference learning to capture statistical regularities in scanpaths, to quantify the differences between scanpaths. Kucharský et al. (2020) used the Scanpath Transition Probability Matrix, which is a matrix that represents the probability of moving from one state, or AOI, to another, as a feature extraction method to transform their scanpaths. Finally, Pejić et al. (2021) used the Sequence Graph Transform (SGT) algorithm (Ranjan et al., 2022), which is a feature embedding function commonly used in data mining. The majority of these methods are a bit complex, but they provide a single algorithm to transform the scanpaths before the learning process. The only outlier was Lethaus et al. (2013), who used a simple feature, i.e., fixation duration within each AOI, as their single feature of interest.

#### 4.2.2 Data transformation

Data transformation is the final step in preparing scanpath data. It focuses on splitting the data into different subsets for training and testing. This is crucial to ensure that ML models perform well and generalize to unseen data. One common method for data splitting is the holdout method, where the dataset is divided into training, testing, and, sometimes, validation subsets. The training subset is used to train the model, the testing subset is used to evaluate its performance, and the validation subset is used to fine-tune the model parameters. There are various strategies for performing the holdout split. We found that the most commonly used method was to use separate datasets or sessions (*n* = 9 Experiments), where complete sessions or datasets are used for training, and the remaining sessions or different datasets are used for testing. Without a validation subset, an 80-20 split (*n* = 7 Experiments) and a 50-50 split (*n* = 5 Experiments) were the most common training-testing splits. However, with the addition of a validation subset, a 70-20-10 split was the most common training-testing-validation split. User-dependent (*n* = 10 Experiments) is a special case where each user's data is split into training and testing subsets to create a unique model for each user.

Resampling is another approach that encompasses cross-validation and bootstrap resampling. Bootstrap resampling was only used by Nikolaides et al. (2016) to generate multiple random replicas of the original dataset for training and testing the model, but it replaces the selected samples back into the original dataset once again, allowing it to be picked multiple times. By generating multiple smaller subsets, they are able to estimate the distribution of the model performance afterwards. On the other hand, cross-validation, which encompasses multiple strategies, was a lot more common. K-fold Cross-validation (*n* = 25 Experiments) works by splitting the data into *K* equal-sized groups where *K*−1 groups are used for training the model, and one group is used for testing the model; this is then repeated *K* times (*K*-fold) to ensure that each group has been used in the training and testing. From the publications that reported the value of *K*, we found multiple options, with *K* = 10 being the most common, followed by *K* = 5. Leave-one-out Cross-validation (*n* = 25 Experiments) is a special type of cross-validation where instead of dividing the data into K groups, one sample or stimulus is used for testing the model and the remaining samples or stimuli are used for training the model; this is then repeated over the full dataset to ensure that each sample or stimulus has been used in testing the model. Another type of cross-validation is Leave-user(s)-out Cross-validation (*n* = 24 Experiments), where the full data from a single user or a group of users are used for testing the model, while the data from the remaining users are used to train the model, which is then repeated for all users; this ensures that the data belonging to a certain user appears either in the training or the testing subsets and not in both.

We can see that Cross-validation accounts for the majority of the reported ML experiments, making it the most preferable method in the literature we reviewed. Cross-validation is more computationally expensive than the holdout method. However, training and testing the model on multiple different data splits provides a better indication of the model's performance.

### 4.3 Learning process

The learning process is the last step in the ML workflow. At this point, the learning task should have been well-defined, the data curation should have been finished, either by using an already available dataset or conducting a user study to collect a new dataset, and the learning data preparation to decide on the scanpath representation format, the scanpath features, and the data split should have all been done. A lot of these decisions are closely related to the learning step. This section discusses the commonly used ML algorithms for scanpath processing, the commonly reported best-performing models, and the most commonly used evaluation metrics. In addition, we focus more on the neural networks due to their complexity.

#### 4.3.1 Model selection

ML algorithms are often split into three main categories: supervised learning models, unsupervised learning models, and reinforcement learning (RL) models. **Supervised learning** is when we have a labeled dataset. It can be used for classification, where we predict a discrete value, e.g., gender, or regression, where we predict a continuous value, e.g., stock prices. **Unsupervised Learning** is when we have an unlabeled dataset. It is often used for dimensionality reduction or clustering problems. **Reinforcement Learning** is a different type of problem where a model tries to learn how to behave through trial and error (Kaelbling et al., 1996), e.g., autonomous driving.

In our reviewed papers, we had 67 publications that used supervised learning models, 24 that used unsupervised learning models, and only one publication by Jiang et al. (2016) that used the Least-Squares Policy Iteration (LSPI) RL. We found that publications tested more than one algorithm on average before deciding on the best-performing one. Supervised learning publications tested, on average, two models (2.075 ± 1.636). Some publications evaluated only one model, while others evaluated up to six (Roy et al., 2020; Krishnan et al., 2021) or seven (Lanatà et al., 2013; Ktistakis et al., 2022) models. However, unsupervised learning publications tended to focus, on average, on just one model (1.304 ± 0.765).

##### 4.3.1.1 Supervised learning

For supervised learning problems, we found a total of 43 different models, which we grouped into 12 categories: Bayesian Algorithms, CNN-based Architectures, Decision Trees, Ensemble Models (Random forest, Bagged Tree, Gradient Boosting, XGBoost, Adaboost, Gradient Boosted Decision Trees, Decision jungle), Linear Models (Linear Regression, Logistic Regression, Lasso, Multiple Regression), Linear or Quadratic Discriminant Analysis (LDA & QDA), Multilayer Perceptron (MLP), Nearest Neighbors, Gaussian Processes, Recurrent Neural Networks (RNNs), Support Vector Machines (SVMs), and Other Neural Networks which includes networks that were not specified or that did not fall into either CNN-based, RNNs, or MLP such as Feedforward–backpropagation (FFBP) network, Probabilistic Neural Networks (PNNs), Radial Basis Function Neural Networks, and Kohonen Self-Organizing Map.

**Figure 9:**
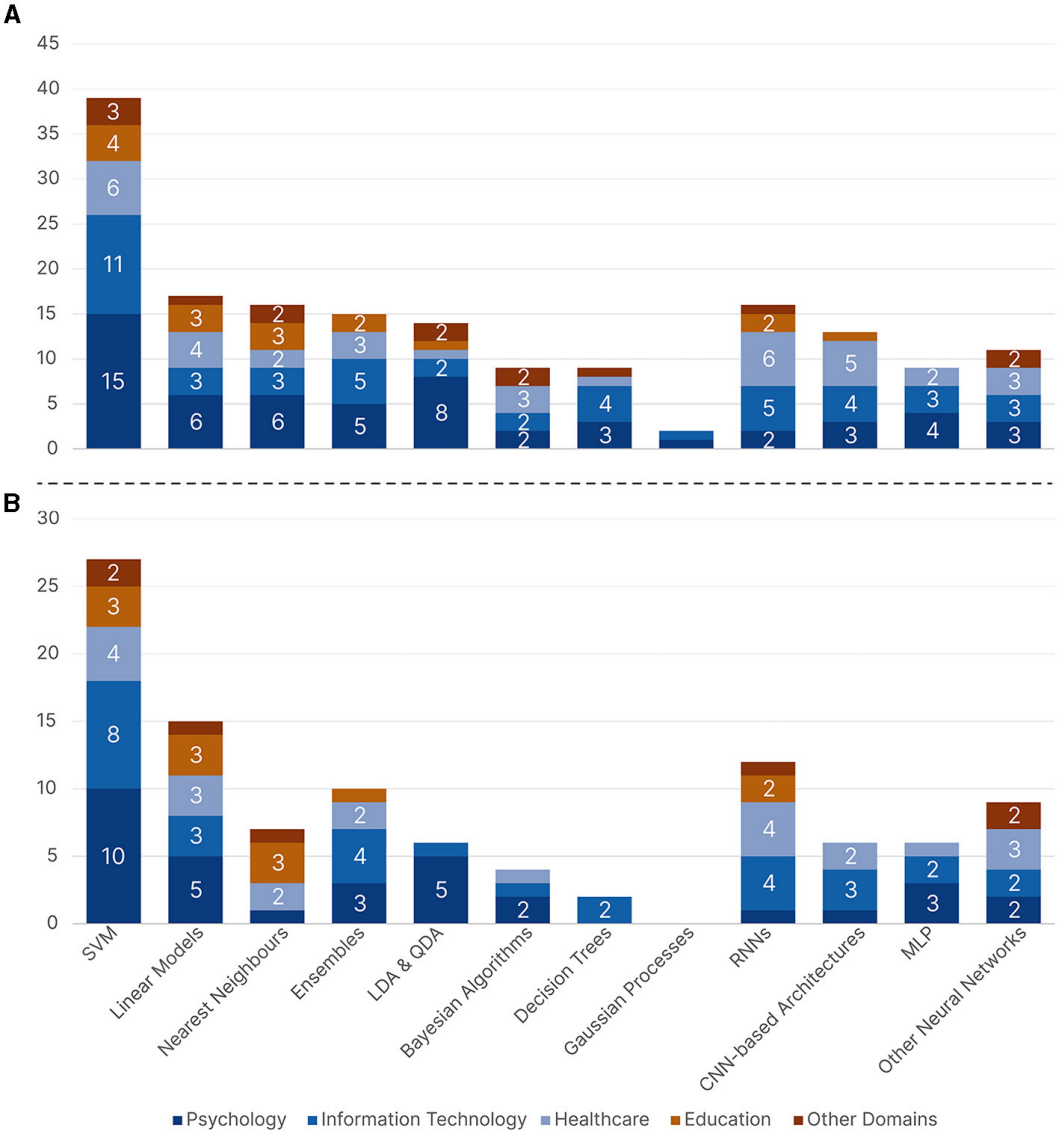
A bar chart showing the supervised machine learning algorithms across all five research domains where **(A)** contains all the reported algorithms and **(B)** contains the reported best-performing algorithms.

Figure 9A shows the distribution of the reported supervised learning algorithms with respect to the five research domains. SVM models were the overall most common across all five research domains, except for *Healthcare*, which had RNNs tied with SVMs as the most common models. Almost all research domains were more inclined toward more traditional ML algorithms as opposed to neural networks. When looking at traditional ML algorithms aside from SVMs, *Information Technology* used Ensemble and Decision Tree models, which are models that usually require hyperparameter optimization, more often than other domains. While both *Psychology* and *Education* used simple models such as Linear, Nearest Neighbors, and LDA & QDA models. When focusing on neural networks, *Healthcare* and *Information Technology* were the two domains with increased neural network usage, as opposed to *Education*, which was the domain with the least usage of neural networks. Figure 9B shows the distribution of the best-performing supervised learning algorithms with respect to the five research domains. The distribution did not change much; SVM still came on top as the most commonly reported best-performing model, followed by Linear models and RNNs, respectively. However, Decision Trees, Bayesian, and Gaussian models were less likely to be chosen as the best-performing models.

##### 4.3.1.2 Unsupervised learning

Twenty-four out of the 77 reviewed publications used unsupervised ML algorithms. They were used for clustering, dimensionality reduction, feature selection, feature generation using Autoencoders, which were only used by Xia et al. (2021), and data generation using Generative Adversarial Networks (GANs), which were only used by Fuhl et al. (2019). We found four types of clustering algorithms: Partitioning Clustering (*K*-Means, Fuzzy *K*-Means), Density-Based Clustering (DBSCAN), Hierarchical Clustering, and Grid-Based Clustering (BIRCH). In addition, we found two types of dimensionality reduction and feature selection algorithms: Principal Component Analysis (PCA), and Manifold Learning which includes the Isomap Algorithm used by Chaby et al. (2017), and t-distributed Stochastic Neighbor Embedding (t-SNE) used by Król and Król (2019).

**Figure 10:**
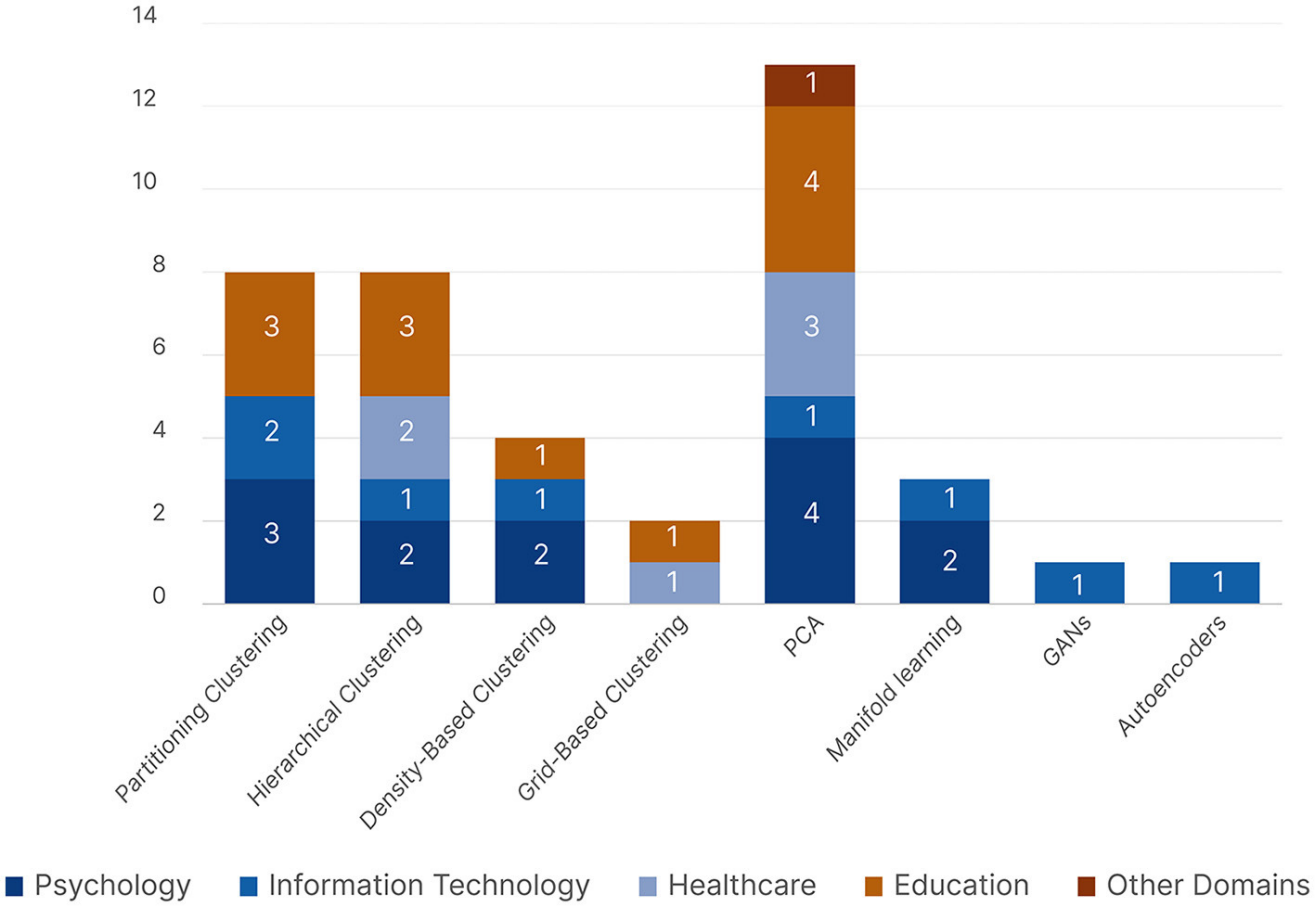
A bar chart showing all the reported unsupervised machine learning algorithms across all five research domains.

Figure 10 shows the distribution of the unsupervised learning algorithms with respect to the five research domains. *Psychology* and *Education* were the main research domains to use unsupervised learning. PCA was the most common unsupervised learning algorithm and the only one used by all five research domains. We can see that both Partitioning and Hierarchical Clustering were more popular than Density-Based and Grid-Based Clustering. *Information Technology*, represented by Fuhl et al. (2019); Xia et al. (2021), was the only domain to use unsupervised neural networks.

#### 4.3.2 Neural network insights

Artificial neural networks are a subset of ML that are inspired to simulate neurons similar to a human brain (Krogh, 2008). Neural networks are often more complex than traditional ML algorithms and often require larger datasets and fine-tuning. In order to provide a comprehensive overview of how ML is used in scanpath analysis for passive gaze-based interaction, we focus in this section on the different details related to neural networks reported across the reviewed publications. We start with the preprocessing steps, which are the same for traditional ML algorithms as well, then the network hyperparameters, and finally, the commonly followed network architectures.

##### 4.3.2.1 Preprocessing

Across the publications that reported any preprocessing, we found that there were seven main preprocessing groups: Artifact Removal, Data Scaling, Data Augmentation, Coordinate Transformation, Windowing, Image Processing, and Fixation Filtering.

**Artifact Removal** involves removing any unwanted data points, such as blinks, noise, or outliers. Most publications reported using the eye tracking manufacturer's software for blink detection and removal. However, it was different for other artifacts. For example, Alghofaili et al. (2019) used a Moving Average filter, and George and Routray (2016) used a Savitzky-Golay filter to remove noise, while Lan et al. (2020) used a Sliding Window Median filter to remove outliers. **Data Scaling** consists of data standardization, which re-scales the data to have a mean of zero and a standard deviation of one, and data normalization, which transforms the data to the same scale to ensure uniformity; for example, the minimum value and the maximum value get transformed into zero and one, respectively, and every intermediate value gets transformed into a decimal value between zero and one. **Windowing** refers to sliding windows that split continuous data into smaller subsets, or expanding windows, which add or expand a window with new values over time. **Data Augmentation** is a technique to increase the data size artificially. This can be achieved by rotating or flipping existing images, resampling existing data, or interpolating new values between existing ones, e.g., SMOTE (Chawla et al., 2002) is an interpolation-based technique to increase the size of the minority class. We also considered Zero-Padding as a form of data augmentation because it is used to create equal-sized time series samples or windows by augmenting them with zeros. **Coordinate Transformation** involves changing the data coordinate system. **Image Processing** encompasses a group of four different processes. *Gray Scaling* converts an image to different gray tones ranging between black and white. *Color Coding* encodes different temporal or spatial information, e.g., fixation duration or saccade direction, as different colors or shades when constructing scanpaths. *Image Rescaling* changes the size of an image while preserving the content, a common step when using CNNs, e.g., Atyabi et al. (2023) rescaled their images from 1,680 × 1,050 pixels to 100 × 100. *Extracting Image Patches* also changes the size of the input image, but by cropping the image into smaller patches, mostly around fixations or certain AOIs. Finally, **Fixation filtering** involves limiting fixations above or below a certain duration, e.g., George and Routray (2016) limited their fixations to a minimum of 12 ms and a maximum of 100 ms, while David et al. (2019) limited them between 80 and 1,300 ms.

##### 4.3.2.2 Network hyperparameters

A neural network set of hyperparameters depends on multiple factors, such as the size of the dataset, the model complexity, and the learning task. However, we are reporting the different hyperparameters to provide an overview since they are all related to scanpaths processing and passive gaze-based applications.

**Batch Size** determines the number of training samples used in one iteration. It can be optimized by evaluating different values, e.g., Smith et al. (2018) evaluated an MLP with batch sizes of 5, 10, 25, and 50. However, most publications used only one value, 64 and 100 being the most common. **Number of Epochs** refers to the total number of times that a network passes through the entire training dataset during the learning process. The most common values were 50, 100, and 500 epochs, while larger values, e.g., 1,000 and above, were not that common. **Early Stopping** is commonly associated with larger numbers of epochs to prevent the network from overfitting and for computational efficiency by avoiding unnecessary epochs. However, only six publications reported using early stopping. **Regularization** also helps prevent the network from overfitting. Dropout and L2 regularization were the two most common techniques. Dropout ignores randomly selected neurons during training, which helps prevent overfitting by ensuring that the network does not rely too heavily on any single neuron. The values 0.5 and 0.2 were the most commonly reported dropout rates across the different networks. L2 Regularization, sometimes called weight decay, adds a penalty to the loss function based on the magnitude of the neurons' weights. This penalty discourages the learning process from assigning too much importance to any single neuron. **Loss Function** evaluates how well the network was able to model the training data. Binary cross-entropy was by far the most common loss function and is used for binary classification problems. Cross-entropy is used for multi-class classification problems, while MAE and MSE were also reported, and they are typically used for regression problems where the goal is to minimize the difference between the predicted and actual values. **Activation Function** determines the output of the neural network. Sigmoid activation was the most reported function and is used for binary classification problems. Softmax is used for multi-class classification problems. ReLu was also used often because of the publications that used CNNs for feature extraction. Linear and Gaussian activation functions were also reported, and they are used for regression problems. **Learning Rate** determines the step size toward finding the minimum loss value. The most common learning rate was 0.001, followed by 0.1, which was also the largest reported learning rate. Other less common values ranged from 1*e*^−6^ up to 0.05. **Optimization Algorithm** changes the network parameters to reduce the loss, and the Adam optimizer (Kingma and Ba, 2017) was almost exclusively used by all of the publications.

##### 4.3.2.3 Network architectures

For the network architectures, we report them based on whether the network was used for feature extraction or for making a prediction. We start with the networks used for feature extraction. Xia et al. (2021) used the Autoencoder by Krizhevsky and Hinton (2011) to extract features from a dataset comprising 3,378 samples. The Autoencoder had an encoder composed of five fully connected layers, each with sizes of 675, 4,096, 2,048, 1,024, and 512 sequentially. This was followed by a bottleneck layer and, finally, a decoder that mirrored the encoder's structure but in reverse order. For regularization, they applied a weight decay of 0.0002. They evaluated using 50 and 100 epochs and batch sizes of 100 and 200. However, using convolutional layers was more common for feature extraction. Fuhl et al. (2019) used a GAN composed of a generator with multiple convolutional and deconvolutional layers, and a discriminator with convolutional layers to convert 5,000 scanpaths into emojis. They used the Cycle Consistency Loss function and a batch size of one. Chung et al. (2018), Castner et al. (2020), and Kanhirakadavath and Chandran (2022) all used CNNs for their feature extraction. All four publications, i.e., Chung et al. (2018); Fuhl et al. (2019); Castner et al. (2020), and Kanhirakadavath and Chandran (2022) essentially followed a similar structure by having multiple convolutional layers, each followed by a mixture of ReLu activation, Max Pooling, or Batch Normalization. They all used Dropout functions and Fully Connected layers, aside from Castner et al. (2020), who used a VGG-16 (Simonyan and Zisserman, 2015) network pre-trained on the ImageNet dataset (Deng et al., 2009), but without a Dropout or a Fully Connected layer. Li et al. (2021) were the only ones to use a combination of convolutional and recurrent layers in their framework called OBF. OBF consisted of an encoder and four decoders. The encoder had a single convolutional layer followed by a Leaky ReLu, Average Pooling, and two Gated Recurrent Units (GRUs) (Cho et al., 2014). Each decoder had two GRUs followed by Batch Normalization, Sigmoid Activation, and a Contrastive Learning Siamese Network, which is a neural network that encodes two scanpath segments using identical subnetworks to pull similar scanpath segments closer in the feature space and pushes dissimilar ones apart.

Using neural networks for predictions was more common. Lethaus et al. (2013) and Li et al. (2022) used shallow ANNs, with Li et al. (2022) using a linear activation function and Bayesian regularization. Benson et al. (2012) used a PNN, and George and Routray (2016) and French et al. (2017) used RBF and FFBP Networks, respectively. However, RNNs, CNNs, and MLPs were much more common. For the reported RNNs, all of them used LSTMs (Hochreiter and Schmidhuber, 1997) aside from David et al. (2019) who used GRUs with a Gumbel Softmax activation followed by Log Transformation. The reported LSTM networks usually had either one or two layers, each followed by a mixture of Batch Normalization, Dropout, and Sigmoid activation, before final fully connected layers. Liaqat et al. (2021) were the only exception with five LSTM layers. All of the used LSTMs used an internal Tangent activation function. The publications used different LSTM sizes, but 512 (Alghofaili et al., 2019), 200 (Xia et al., 2021; Castner et al., 2022), and 128 (Chung et al., 2018; Xu et al., 2018) were common values. The reported CNNs were quite similar, consisting of a mixture of two to four convolutional layers, each followed by a mixture of ReLu, Dropout, and Max Pooling functions, then fully connected layers before the final activation function. The only exceptions were Atyabi et al. (2023), who used 32 convolutional layers, and Liaqat et al. (2021), who used a pre-trained ResNet-18 (He et al., 2015). The VTNet from Sims and Conati (2020) was also interesting because they combined a CNN that processed scanpath images in parallel to a GRU that processed the raw gaze data. The 2-layer CNN and the 1-layer GRU with 256 units were combined afterwards using two fully connected layers and a Softmax activation. The reported MLPs were not often provided with a lot of details. Smith et al. (2018), Li et al. (2021), and Liaqat et al. (2021), all reported similar architectures of using two or three layers each followed by ReLu, Batch Normalization, and Dropout, before a final fully connected layer.

#### 4.3.3 Model evaluation

Across all 77 publications, multiple publications reported multiple model evaluation metrics. However, they all focused on a specific metric either in the abstracts, discussions, or conclusions, so we noted the main metrics from the different publications. Accuracy was the choice of most publications, as it was reported 44 times, followed by Area under the ROC Curve (AUC) score being reported seven times. Other notable mentions are the F1 score (*n* = 5) and Recall, i.e., True Positive Rate (TPR) (*n* = 4). Error rates were also used, especially for evaluating clustering algorithms, which include Mean Absolute Error (MAE), Mean Squared Error (MSE), Normalized Root Mean Squared Error, and Root Mean Squared Error.

## 5 Discussion

In this review, we presented an overview of the current state of using ML for processing scanpaths in passive gaze-based applications extracted from 77 publications from 2012 to 2022. The overview consisted of two main parts: the first focused on the possible research domains and learning tasks, while the second focused on the different stages of the ML workflow, shown in Figure 6. In this section, we discuss both parts to answer the questions raised in the introduction.

### 5.1 Q1. What research domains have used machine learning to analyse scanpaths, and what passive gaze-based learning tasks have they focused on?

Based on the specific ML task for which the scanpaths were analyzed, we categorized the publications under two dimensions: research domains and learning tasks. We had a total of five research domains and 11 learning tasks, and each publication was categorized under at least one domain and one learning task. The five research domains were: *Education, Healthcare, Psychology, Information Technology*, and *Other Domains*. *Other Domains* includes Driving, Aviation, Maritime, Marketing and Product Design, and Linguistics, but they each had less than three publications, so we combined them under one group. The 11 learning tasks were: *Activity, or Stimuli Inference, Experience, Gender, or Age Categorization, Attention Monitoring, Emotion, or Face Perception/Recognition, Reading, Cognitive Modeling, Psychiatric, Mood, Learning, or Neurodevelopmental Disorders, Medical Decision Support System, User Identification, Gaze Prediction*, and *Other Tasks*. *Other Tasks* includes Security and Privacy, Product Design Evaluation, User Experience Evaluation, and Visual Search, but they each had less than three publications, so we combined them under one group.

**Figure 11:**
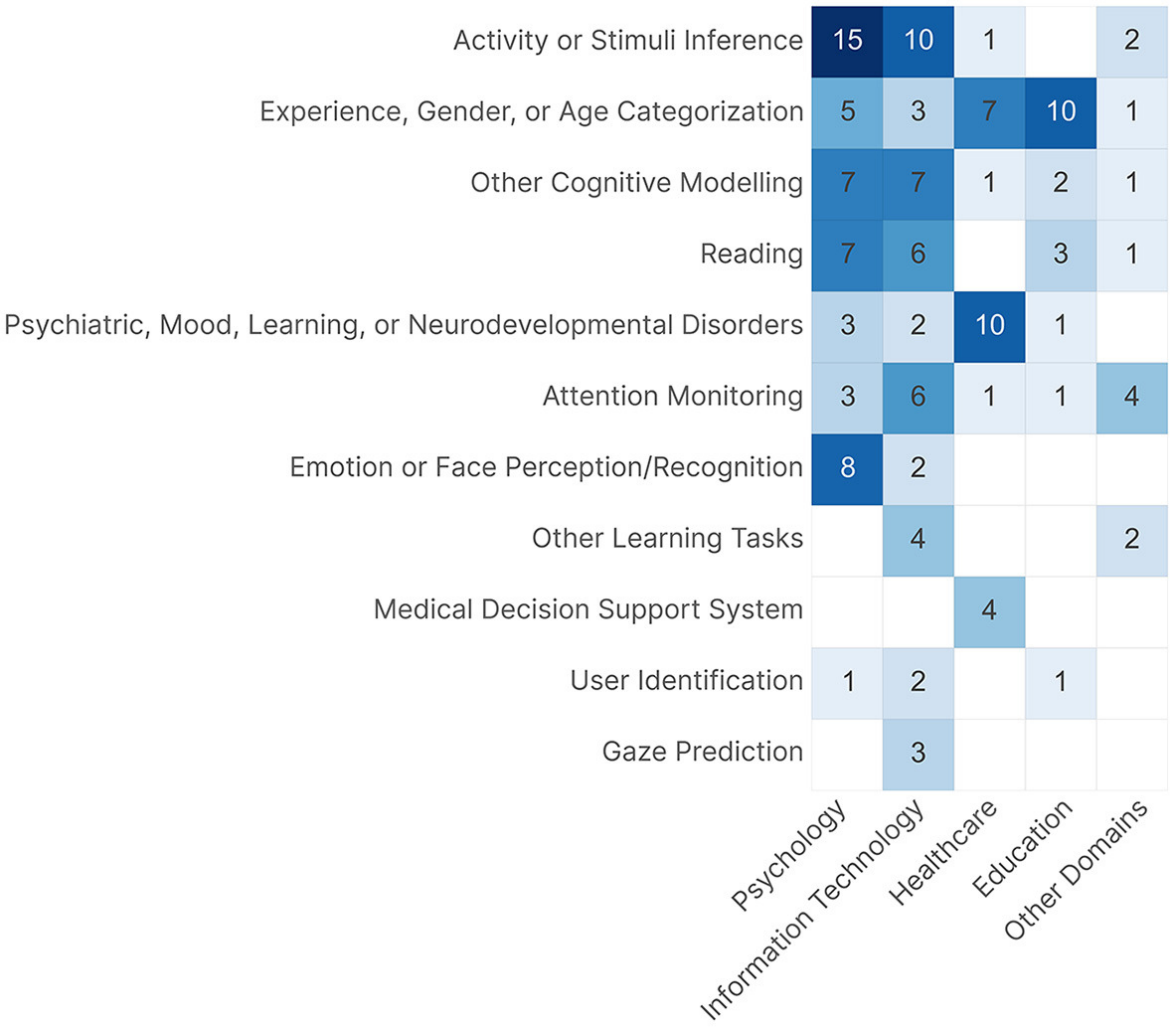
A heatmap showing the distribution of the different tasks within each research domain.

Looking at each dimension separately does not provide the full picture of the research landscape. Figure 11 shows a heatmap of the intersection and distribution of the learning tasks across the research domains. We see that each domain has one or two learning tasks where most of the contribution was focused. The majority of *Psychology* publications focused on **Activity, or Stimuli Inference** tasks, with 15 out of 19 **Activity, or Stimuli Inference** publications being psychology-related, while the remaining four publications, i.e., Lethaus et al. (2013); Srivastava et al. (2018); Fuhl et al. (2019), and Peysakhovich et al. (2022) focused on predicting the task or stimuli without further investigation of the underlying visual behavior. *Information Technology* was the only research domain seen across all the learning tasks, except for **Medical Decision Support System**. We found that multiple publications focused more on the technological implementation (*n* = 15 Publications) for different learning tasks and only used the task as a way to evaluate the technology, which is why we categorized these publications under *Information Technology*; this caused it to have a large intersection with other research domains across the different learning tasks. Both *Information Technology* and *Psychology* show quite similar behavior in the distribution of a few learning tasks, mainly caused by publications on Human-Computer Interaction (HCI); this can be seen in **Activity, or Stimuli Inference**, which was also the most common learning task within *Information Technology*, and in **Other Cognitive Modeling** and **Reading**. *Healthcare* had all the **Medical Decision Support System** publications, a large number of **Experience, Gender, or Age Categorization** publications due to the focus on healthcare professionals' visual behavior, and all of **Psychiatric, Mood, Learning, or Neurodevelopmental Disorders** publications except for Król and Król (2019) because they focused on understanding the difference between ASD and Typically Developing (TD) children in their face-scanning patterns, and not on pure diagnosis. We initially expected **Psychiatric, Mood, Learning, or Neurodevelopmental Disorders** publications to be fully categorized under *Psychology*; however, most of them did not focus on studying the actual underlying visual behavior or cognitive process associated with each task, but rather on the diagnosis. *Education* had the majority of **Experience, Gender, or Age Categorization** publications; this is because most of the publications focused on differentiating between different experience levels to provide insights to improve the learning process. Four out of the seven publications categorized under *Other Domains* focused on **Attention Monitoring**.

Our two-dimensional categorization is quite similar to the approach followed in the seminal research work of Duchowski (2002), but with a few key differences. Regarding the research domains, we did not encounter neuroscience publications, i.e., publications that focus on the neural components of vision. We had three publications that used eye tracking alongside EEG devices (Shi et al., 2017; Giannakos et al., 2019; Moon et al., 2021), but they did not qualify as such because they focused on various tasks and not on how the changes in one signal translated to the other signal and so on. In addition, Duchowski (2002) had *Industrial Engineering and Human Factors* as a domain, but we split it into its components of *Aviation* and *Driving*. Lastly, we did not want to name a domain as *Computer Science* because all of our publications fall under computer science with their focus on ML; we opted for *Information Technology* as a substitute to avoid causing confusion. Duchowski (2002) stated that broader applications will emerge with the improvement in computer and graphical systems. This statement holds true and can be seen in our categorization of more than eleven distinct passive gaze-based applications across our review. Our tasks differ from their task categorization because we focused on what the authors wanted to infer from the ML algorithms and not the actual visual activity the participants were undergoing; for example, publications on Scene Perception were mostly categorized under **Activity, or Stimuli Inference** or **Attention Monitoring**. However, we had a lot in common, such as reading and visual search tasks.

### 5.2 Q2. Which research domains and passive gaze-based learning tasks have yet to use machine learning in scanpath processing?

After identifying the research domains and learning tasks, we saw that there were research domains that have yet to use ML in scanpath processing for passive gaze-based learning tasks. This section highlights some domains and tasks that we think could warrant more focus in future research.

Regarding the research domains, *Sports* is an interesting domain for eye tracking to understand decision-making for both players and referees, but surprisingly, despite finding sport-related publications in our initial pool, none of them used ML to process scanpaths. *Gaming* is also quite popular as an important application domain, with certain eye trackers being marketed toward gaming purposes,^14^ but we were unable to find hits, even in the initial pool of publications. Aside from completely missing domains, we expected to find more publications on *Linguistics*, but we were only left with just one publication. We can see that a lot of domains still have a large room for exploration where ML processing of scanpaths might be a suitable solution. Multidisciplinary publications, i.e., publications categorized under more than one domain, could also be a promising research direction. The UpSet plot in Figure 4 shows that 52 publications out of 77 were categorized under just one domain. This can be further supported by the fact that most of the remaining 25 publications had an intersection with *Information Technology* due to focusing mainly on the technology rather than the task, which left even fewer pure multidisciplinary publications. *Education* and *Healthcare* had a total of six common publications, which was due to **Experience, Gender, or Age Categorization** publications that focused on how different levels of experience affect the visual behavior of healthcare professionals. This opens two possible future research directions, one direction where the learning task could be utilized in exploring the differences between professionals in other domains aside from *Healthcare* to see if the established findings can be generalized and transferred to other professions. The other direction focuses on exploring different learning tasks in the *Education* domain as it still has a large room for improvement given that it was the research domain with the least diversity.

Regarding the learning tasks, **Evaluating Product Design and User Experience** only had two publications despite being an active area for scanpath analysis in general. This could mean they still depend on manual evaluation without ML automation to infer insights. Extended Reality (XR) publications were also not as common as we had initially expected, especially with the advances in headsets with integrated eye tracking, which increased the popularity of their intersection (Plopski et al., 2022). We only had three publications that used Virtual Reality (VR). Alghofaili et al. (2019) focused on predicting if a participant needed help in navigating a virtual environment, Kim et al. (2022) focused on stress and cognitive load monitoring, and Xu et al. (2018) focused on scanpath prediction. Scanpaths have the benefit of retaining temporal information; if the order of actions is important, then scanpaths offer valuable insights. We believe that the intersection of XR applications, both VR and Augmented Reality (AR), and scanpath processing could open opportunities for both domains and tasks; it also presents opportunities for research into solving technical challenges regarding adapting current efforts to 3D setups. There are already multiple available toolkits that could help in this regard. Kapp et al. (2021) presented ARETT, a toolkit for reliable eye tracking data acquisition in AR; it was later tested in visual Attention Monitoring and identifying the objects in a user's view in an AR setup (Barz et al., 2021b). Evangelista Belo et al. (2022) presented AUIT, a toolkit for designing adaptive user interfaces (UIs) in XR, which can be extended to work with gaze data. Creating adaptive UIs is an interesting research area because it can make use of ML algorithms that use various passive gaze-based cues to change the UI in response. For example, any of the tasks in **Other Cognitive Monitoring**, e.g., stress, can be a trigger to change the UI in a way to facilitate the user's experience; or **Experience, Age, or Gender Categorization** tasks, e.g., detecting the age group of the user to present a different UI to children, teenagers, adults, and seniors in a way to keep them safe and offer them a tailored experience. This shows that certain tasks still have room for further investigation and that other tasks are more mature, which also allows for investigating novel ML methods since you could compare them against already established benchmarks.

### 5.3 Q3. What are the commonly followed ML practices, in line with a general ML workflow, that have been used for scanpath analysis in passive gaze-based applications?

We present our answer to this question following our general workflow shown in Figure 6. For each section, i.e., data curation, learning data preparation, and learning process, we summarize the key takeaways that can be used as guidelines for future investigations.

#### 5.3.1 Data curation

For the data curation, we focused on three main points: the eye tracker frequency, the number of study participants, and the participants' demographic information. We found that the majority of publications, i.e., 59 out of 77, preferred to collect their own datasets. However, some publications used already available datasets, which we summarized in Table 1.

Across all the reported datasets, we found that 60 Hz was the most frequently used eye tracking frequency, followed by 250 and 1,000 Hz. Using frequencies <60 Hz was not that common, with only 25% of datasets using frequencies <60 Hz. Eye trackers operating at lower frequencies are often less expensive, but they could lead to sampling errors and not being able to compute certain features accurately. Andersson et al. (2010) argued that sampling errors can be mitigated by collecting more samples but cease to be a problem for frequencies above 200 Hz. Analysis of fixations becomes more stable from 60 Hz, but to correctly detect and analyze saccades, the eye tracker should have at least a 120 Hz sampling frequency (Leube et al., 2017), while some saccade-based features would require frequencies above 200 Hz (Andersson et al., 2010). Sixty Hz might be the most common due to being more affordable, but to avoid these issues, we would argue that an eye tracker of 250 Hz would be better to avoid sampling errors and if we needed to study fast eye movements and saccade-based features.

For the number of participants, we found that collecting data from up to 40 participants was the most frequent strategy, with 24 participants being the most popular choice. However, *Healthcare* publications preferred larger datasets with up to 80 participants. We cannot provide a one-size-fits-all answer because it depends on the number of collected samples, the research question, the study design, and additional factors such as available time and funding. The rule that is mostly followed for eye tracking studies related to usability testing is to collect data from at least 30 participants (Eraslan et al., 2016), but similar statements cannot be made across the full landscape. The provided numbers are just an overview of the field of what is commonly found across the literature, but for a more informed decision to compute the number of participants, we would recommend reading publications such as (Martínez-Mesa et al., 2014; Brysbaert, 2019). In addition, the Supplementary material has full details for more detailed insights toward the number of participants used by each domain or learning task.

We found inconsistencies in reporting demographic information across the reviewed literature, such as the participants' genders, nationalities, and ages. Reporting this information could benefit future use of the datasets, e.g., focusing on use cases for certain genders or age groups. We recommend using checklists such as the one from Dunn et al. (2023), which provides a list of information that should be included when reporting eye tracking studies and the reasons behind their inclusion.

#### 5.3.2 Learning data preparation

For the learning data preparation, we focused on two main points: how scanpaths are formatted as suitable inputs to an ML algorithm and how the data is split into training, testing, and, sometimes, validation subsets.

We found that there were six main formats for representing scanpaths as suitable inputs to an ML algorithm: Visual Encoding, String Representation, Time Series, Graph Representation, Feature Engineering, and HMM. We referred to these six methods as scanpath representation formats because publications presented the scanpath data as one or multiple of these formats, which was then fed into the ML algorithm. Feature engineering was the most preferred format, followed by string representation. Aside from feature engineering, *Psychology* publications preferred to use HMMs and time series representations. This might be due to using stimuli without explicit AOIs or stimuli with different layouts, where the visual behavior might be a bit complex, or due to the importance of the temporal information in their learning tasks since HMMs are often used to encode temporal data. *Healthcare* publications preferred to use visual encoding formats, which might be due to their focus on image-based stimuli. *Education* publications preferred string representation formats. String representation focuses on the transitions between the AOIs within the stimuli in order to enable a quantifiable comparison between pairs of scanpaths; they are often used to compare pairs of scanpaths, especially for group categorization, which aligns with the **Experience, Gender, or Age Categorization** learning task. Lastly, *Information Technology* publications preferred both time series and string representations.

For feature engineering, we found that publications used five features on average (5 ± 5.965). We found a total of 126 different features extracted from scanpath data. Saccade-based features were the most diverse, but fixation-based features were more commonly used, especially average fixation duration, total fixation duration, and total fixation count, while scanpath length was the most commonly used full scanpath feature. The Supplementary material provides the full list of features, our feature categorization, and the mapping to their respective publications, research domains, and learning tasks. To tackle a specific learning task, we think it is better to establish a baseline by using the features commonly used by other publications to solve the task and then explore using additional feature combinations that are not commonly explored within this task. Some features, such as scanpath spatial density and saccade duration per AOI, which were used by only one publication each, could also be worth investigating. Based on the findings, we think using five to ten features is a good starting point, but caution is needed to not overfit the ML model by using many features (Ying, 2019). In case of needing to use just one feature extraction method, scanpath comparison algorithms, neural network computed features, scanpath transition probability matrix, the SRSA algorithm (Hayes et al., 2011, 2015), the SGT algorithm (Ranjan et al., 2022), the OBF framework (Li et al., 2021), or fixation duration within each AOI might be the best starting points.

Finally, for the data splits, publications preferred cross-validation as opposed to holdout methods despite being more computationally expensive. *K*-fold Cross-validation has been found to provide a better model evaluation estimate and a better generalizability estimate than holdout (Blum et al., 1999). When using *K* = 1, this is often called Leave-one-out Cross-validation, which maximizes the training data and might be beneficial for small datasets. Leave-one-out is less likely to provide a biased estimate of the model performance compared to larger *K*-values (Fushiki, 2011), but it is very computationally expensive for large datasets. The choice of *K* is very critical; based on the reviewed literature, *K* = 10 and *K* = 5 might be the best starting points. A different approach called Leave-user(s)-out Cross-validation is very useful for user-independent use cases where we would like the method to generalize across different users. Leave-user(s)-out provides better estimates of the model generalizability to unseen participants' data (Cho, 2021); with some authors arguing that for physiological data, such as eye tracking data, *K*-fold might overestimate the model performance and should be evaluated using Leave-user(s)-out instead (Dehghani et al., 2019; Cho, 2021). It depends a lot on the dataset and the research questions in mind, but Cross-validation seems to be the preferred method across the community, with multiple opinions arguing that Leave-user(s)-out Cross-validation provides better model evaluation estimates.

#### 5.3.3 Learning process

For the learning process, we focused on two main points: the used ML algorithms, with a focus on neural networks, and how they are evaluated.

##### 5.3.3.1 Model selection

We found that supervised ML was more common than unsupervised and reinforcement learning. Publications tended to test, on average, two supervised learning algorithms (2.075 ± 1.636) but only one unsupervised learning algorithm (1.304 ± 0.765). SVM was the most common ML algorithm for supervised learning tasks, followed by Linear Models and RNNs. PCA was the only algorithm that all five research domains used for unsupervised learning tasks, and partitioning and hierarchical clustering were the two preferred clustering methods. We only had one publication for RL, i.e., Jiang et al. (2016), which used the LSPI policy.

**Figure 12:**
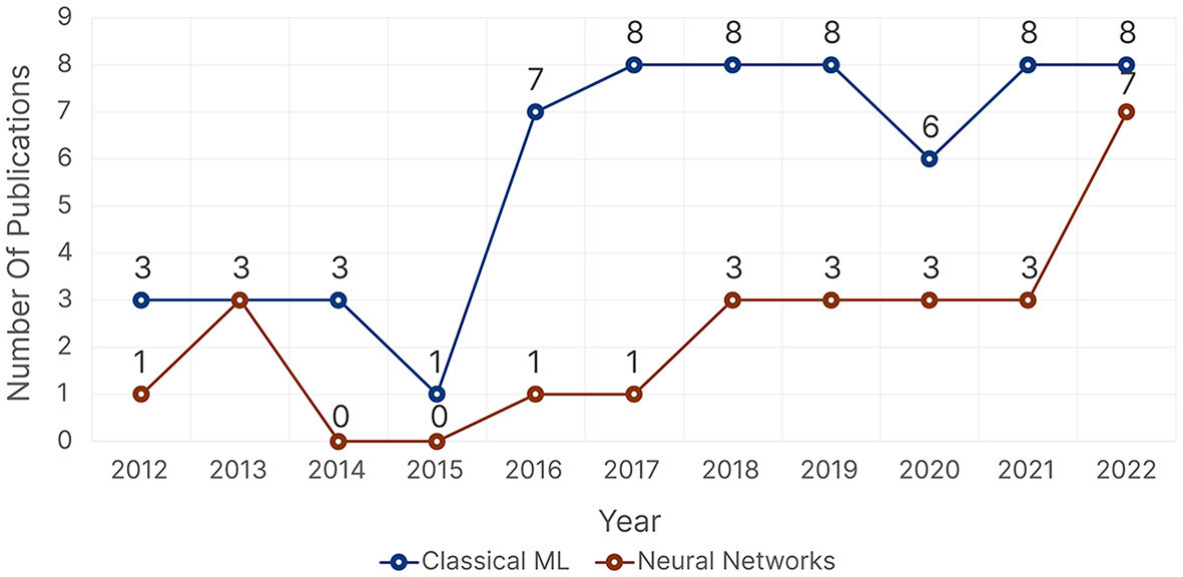
The distribution of traditional machine learning and neural networks per publication for scanpath analysis in passive gaze-based applications from 2012 to 2022.

We found across the different publications that traditional ML was more prevalent than neural networks. This was very clear with unsupervised learning tasks, with GANs and Autoencoders being the only unsupervised neural networks used by Fuhl et al. (2019) and Xia et al. (2021), respectively. Figure 12 shows a distribution of the number of publications in our review that used traditional ML and neural networks across the period of 2012–2022. We can see that traditional ML has always been the preferred option. The gap was quite wide from 2014 onwards, but in 2022, we can see that the number of neural network approaches largely increased. This might mean that moving forward, we might expect more inclination toward adapting neural networks.

##### 5.3.3.2 Neural network insights

To build upon our reported findings regarding neural networks (Section 4.3.2), we looked at relevant publications that were not part of the reviewed papers, and more recent publications from 2023 to 2024. We did not find any differences regarding pre-processing and hyperparameters but rather a few differences regarding the networks themselves.

The studies by Bao and Chen (2020), Kerkouri et al. (2022), and Kümmerer et al. (2022) all used the SALICON dataset (Jiang et al., 2015) to train their networks. Bao and Chen (2020) and Kerkouri et al. (2022) trained a ResNet-50, and a MobileNet (Sandler et al., 2018) with an additional CNN and MLP networks, respectively, to predict visual attention based on saliency maps. Kümmerer et al. (2022) trained a DenseNet201 (Huang et al., 2017) with two additional 2-layer convolutional networks to predict upcoming fixations from prior ones. Bao and Chen (2020) additionally trained a VGG-16 on the OSIE, the KTH (Kootstra et al., 2011), and the EyeCrowd (Jiang et al., 2014) datasets to predict the fixation duration. The studies by Barz et al. (2020), Bhattacharya et al. (2020b), and Barz and Sonntag (2021) are more related to our scope. Barz and Sonntag (2021) evaluated a ResNet-152 pre-trained on the ImageNet (Deng et al., 2009) dataset, and an R-CNN (He et al., 2017) pre-trained on the MS COCO (Lin et al., 2015) dataset, separately, to map visual attention to an AOI. Barz et al. (2020) used a SegNet (Badrinarayanan et al., 2016) pre-trained on the indoor scenes from the SUN RGB-D dataset (Song et al., 2015), and an AlexNet (Krizhevsky et al., 2012) pre-trained with ImageNet to extract features from scanpaths. Bhattacharya et al. (2020b) used a VGG-19 pre-trained on ImageNet to process scanpaths for perceived relevance estimation. All six publications used pre-trained convolutional models on large image datasets.

Using pre-trained convolutional networks did not change much during the last 2 years. We found that VGGs pre-trained on ImageNet are still popular; Byrne et al. (2023a,b) used VGG-16 and VGG-19, respectively, to predict a user's decision. Fuhl (2024) proposed a feature extraction network based on ResNet-12 and evaluated it on the Doves (Bovik et al., 2009), WherePeopleLook (Judd et al., 2009), and Gaze (Dorr et al., 2010) datasets. However, an interesting change was the usage of transformers in more recent publications.

Transformers are deep learning models that were initially proposed for Natural language processing (NLP) tasks by Vaswani et al. (2017) but have since been extended to other domains. Transformers have two key features: self-attention mechanism and positional encoding. The self-attention mechanism allows the model to weigh the importance of the different parts of an input sequence, regardless of their positional distance from each other. Transformers do not process data sequentially like RNNs, so they use positional encoding to incorporate the location information of the input sequence in the embeddings. Transformers are good for large-scale data as they can process data using parallelization and are more highly scalable than other types of deep neural networks. Jiang et al. (2024a) presented a Transformer-guided RL approach, called EyeFormer, to predict personalized scanpaths across various visual stimuli types. The prediction task is framed as a sequence generation problem, where each predicted fixation point is treated as an action taken by the RL agent. They used the Vision Encoder Transformer model by Dosovitskiy et al. (2021) to process visual information by converting image patches into a sequence of embeddings, capturing essential local and global details for comprehensive context understanding. The Fixation Decoder is a multi-layer Transformer that then predicts the next point in the scanpath by utilizing these embeddings and considering the history of previous fixations. They used the REINFORCE algorithm (Williams, 1992; Rennie et al., 2017) for their RL. To evaluate their system, they used the OSIE and the Ueyes (Jiang et al., 2023, 2024b) datasets. Unger et al. (2024) proposed a Transformer-based deep learning architecture, called RETINA, to predict a user's product preference from raw eye-movement data. They employ a multi-layer bidirectional Transformer architecture, similar to that of Vaswani et al. (2017), to capture intricate temporal relationships between gaze points and predict AOIs by leveraging the Transformer's inherent ability to process long sequential data in parallel.

##### 5.3.3.3 Model evaluation

The evaluation metric depends a lot on the dataset at hand and the type of problem. We found that accuracy was the predominant ML model evaluation metric, followed by AUC score and F1 score, respectively. There are various publications that focus on the benefits and limitations of each evaluation metric; for example, accuracy is not suitable for imbalanced data, and F1 score is not suitable when you care equally about the positive and negative classes. This is why some publications support using alternative metrics; for example Chicco and Jurman (2020) argued that Matthews correlation coefficient (MCC) (Baldi et al., 2000) has benefits over both accuracy and F1 score for binary classification problems. We argue that computing and reporting more than one metric gives a better model evaluation estimate, but the choice of which metric to focus on depends on multiple factors, and publications such as Hossin and Sulaiman (2015); Naidu et al. (2023) help in making an informed decision.

### 5.4 Q4. Are there any notable emerging machine learning topics that have not been investigated for passive gaze-based scanpath processing, and what benefits could they provide?

The field of ML is fast-paced, with new algorithms and approaches emerging on a regular basis. We saw that scanpath processing has a lot of potential for exploring different ML approaches than the current focus. We formulated this question to provide insights toward three ML topics that might help tackle current challenges and open future opportunities that might prove useful in scanpath processing. The three topics are Self-supervised learning, Transformers, and Explainable AI. We start by explaining each topic and offer our opinion on how they can be utilized for scanpath processing in passive gaze-based interaction.

#### 5.4.1 Self-supervised learning

Self-supervised learning (SSL) is an ML approach where the system learns to understand and work with data without being explicitly provided with labeled examples during training; instead, it generates its labels from the input data (Nguyen et al., 2021; Liu et al., 2023), which is achieved by designing a task where the model predicts some parts of the data using other parts of the data. SSL differs from unsupervised learning because instead of dealing with structural patterns in the data for clustering or dimensionality reduction, it focuses on solving supervised learning tasks such as classification, which can be done using multiple approaches.

Pretext learning is an approach which consists of a pretext task and a downstream task. In the pretext task, the model learns generalizable feature representations of the data distribution using labeled data, while in the downstream task, the model transfers its pretext knowledge to a different task with less labeled data. For example, Dubey et al. (2022) had a pretext task of using the relative pupil positions in estimating the gaze direction, i.e., right, left, or center, which was then used for a downstream task of visual Attention Monitoring. In the Contrastive Learning SSL approach, the model is trained to identify similar, i.e., positive, and dissimilar, i.e., negative, pairs of data points; this helps the model to encode the data into a representation space where similar data points are close and dissimilar data points are far apart (Chen et al., 2020).

SSL could be used in multiple tasks such as scanpath generation, experience gender or age categorization, general user grouping, and cross-modal learning. In cross-modal learning, we could use scanpath data accompanied by data from different modalities, such as EEGs or physiological monitoring wristbands. However, it also presents multiple challenges, such as designing appropriate pretext tasks and evaluation methods.

#### 5.4.2 Transformers

As we have seen in Section 5.3, Transformers have recently been used in relevant publications. Transformers have a lot of potential in processing scanpaths because they can take the temporal information, i.e., the order of fixations and saccades, into consideration. Attention mechanisms could also focus on specific parts of a scanpath if we know that a certain behavior or a certain AOI holds more importance than others. These could be an entryway to learning tasks that still depend on manual interpretation, such as usability studies. Another interesting intersection would be to use Large Language Models (LLMs), which are transformer-based models, to investigate how they could potentially help in scanpath analysis. For example, to generate a descriptive narrative of a scanpath behavior with respect to the AOIs, or predict potential AOIs based on previous scanpaths. However, this intersection presents a few challenges. LLMs need to have enough contextual information to interpret scanpaths, model training and optimization would require large computational resources, and the integration is a novel use case, so the implementation itself might be technically challenging.

#### 5.4.3 Explainable AI

Explainable AI (XAI) or explainable methods in ML are techniques designed to help humans understand and trust the decisions made by ML models by shedding light on the reasoning behind model predictions, making the models more transparent and their decisions easier to interpret (Angelov et al., 2021; Kadir et al., 2023). Traditional ML algorithms are generally inherently explainable; for example, Valdunciel et al. (2022) created ReMA, a simple interactive tool designed to assess gaze-based relevance estimation models; it visualizes the stimulus with a heat map for fixation duration alongside the values of the extracted features, model prediction, and ground truth; this enhances the transparency of the model, and allows researchers to better understand the strengths and weaknesses of the model. However, deep neural networks represent the main challenges in XAI because it is not inherently clear how their internal computations were able to reach the given output, and such tools as ReMA might not be as helpful. There are multiple XAI methods, but we will only discuss a few relevant ones.

Model-agnostic methods are used to explain the predictions of any ML algorithm regardless of its type or complexity. For example, Local Interpretable Model-Agnostic Explanation (LIME) Lundberg and Lee (2017) alters an input data point, e.g., changing a feature value, and observes the corresponding changes in the model output to understand how each feature affects the predictions and which features are the most important. The main problem with these methods is that they might not provide good insights into deep neural networks due to their model complexities and abstract feature space. Gradient-based explainable methods, e.g., Grad-CAM (Selvaraju et al., 2017) and Grad-CAM++ (Chattopadhay et al., 2018), are commonly used for explaining deep neural networks. Unlike Model-Agnostic Methods, which are invariant to the type of models, these methods are model-dependent (Samek et al., 2021). Activation Map Methods, e.g., Grad-CAM (Selvaraju et al., 2017) and Saliency Maps (Simonyan et al., 2014), try to understand which regions in the input data activate certain neurons strongly to try and find out the most important regions contributing to the prediction. They mostly work with visual inputs and computer vision tasks to generate heatmaps, i.e., activation maps, that highlight the most important regions in the input image that the model used to make its prediction (Samek et al., 2021). Integrated Gradients (Sundararajan et al., 2017) can be applied to any differential models, making it versatile across many types of neural networks for different data modalities, including eye-tracking. Activation Map methods could be used to explain the model prediction with visual encoding scanpath representation format. Integrated Gradients could be beneficial to use with feature engineering. XAI could help identify the underlying scanpath visual behavior that led to a certain prediction, which could help in better user modeling, it could be used for feature importance and maybe sequence importance in a string representation, and it could be used to understand how different stimuli affect a model prediction in order to understand the causal relationship between scanpaths and stimuli.

### 5.5 Limitations, challenges and ethical considerations

In order to give a full picture of how ML has been used for scanpath analysis in passive gaze-based applications, we also need to discuss the limitations of the current approaches and the ethical considerations of using ML in passive gaze-based applications as a whole.

#### 5.5.1 Limitations and challenges

In this section, we discuss limitations and challenges identified in the reviewed publications. The main reported aspects were real-time processing, efficiency, and effectiveness. In addition, we elaborate further on the reproducibility and replicability of the proposed systems.

##### 5.5.1.1 Real-time

We found that ML methods for scanpath analysis are often limited to offline applications such as in *post-hoc* experiments. The real-time capabilities and related challenges are rarely discussed, if at all, for example the latency of the proposed systems, their throughput, and their hardware constraints. Across the 77 publications, only a few publications, i.e., Biedert et al. (2012); Ishii et al. (2013); Moacdieh and Sarter (2017); Raptis et al. (2017); Alghofaili et al. (2019); Kelton et al. (2019); Fu and Steichen (2022); Southwell et al. (2022), addressed this aspect.

Processing scanpaths in real time poses several challenges. One primary challenge is deciding on suitable preprocessing steps and algorithms. In Section 4.3.2, we presented the different pre-processing steps reported when using neural networks. The reported artifact removal methods necessitate a compromise between adding delays and removing noise, which could affect the overall performance. The reported data scaling methods require knowledge of the statistical properties of the entire dataset, which might not be available in real-time. Windowing is also quite important because using sliding windows would require a trade-off between having sufficient samples for the ML algorithm to function properly and not introducing large time delays, which could affect the overall user experience; while using ever expanding windows would be inefficient. This poses a question regarding whether or not to discard older samples, which can be facilitated using available tools and frameworks, e.g., Barz et al. (2021a).

In Section 4.2.1, we presented the different scanpath representation formats, but the publications did not consider their suitability for real-time processing. For example, deciding whether to use fixations or raw gaze samples to construct a scanpath is crucial. Using fixations in real-time would require using suitable online fixation detection algorithms, e.g., Santini et al. (2016); Lobão-Neto et al. (2022). Otherwise, some detection algorithms are either infeasible or make use of future samples, which can cause delays. While string and graph representation would require real-time fixation-to-AOI mapping, e.g., Barz and Sonntag (2021); Barz et al. (2021b). Similar questions arise for deciding the suitability of the different scanpath features for real-time processing. In addition, using a scanpath as a visual encoding requires the full scanpath which is not suitable in real-time and is a limitation of the majority of proposed neural networks.

##### 5.5.1.2 Efficiency

In Section 4.1.1, we presented the different eye tracker frequencies. The majority of experiments were conducted with lower frequency eye trackers, e.g., 60 Hz. According to the discussion in Section 5.3, lower frequency eye trackers could lead to sampling errors in the recordings, which was not discussed or reported by publications. In addition, we found out that for saccade-based features, it is recommended to use an eye tracker with 120 to 200 Hz, which was also not discussed by multiple publications that used lower frequencies.

The usage of complex machine learning algorithms, especially with large datasets, and cross-validation (Section 4.2.2) can take a lot of resources to train and optimize the model. The specifications of the system that ran the experiments were not always reported. This leads to challenges in estimating the necessary resources, assessing the environmental impact, and determining the cost to train and evaluate the proposed systems (Paleyes et al., 2022). These aspects are often overlooked in existing research literature. We recommend that future studies should at least discuss these aspects in hopes of promoting or engaging in conversations that could potentially lead to solutions for these issues.

##### 5.5.1.3 Effectiveness

Most of the publications in this review reported good performance of their models. However, this does not guarantee that these systems would perform equally well in different settings. In Section 4.2.2 we saw that cross-validation was the preferred option, but holdout was quite common. When using holdout, a claim regarding the system generalizability to other users, tasks, or environments cannot be made. For cross-validation, leaving users, tasks, or stimuli out can prove that the system generalizes well to new users, tasks, and stimuli, respectively. Because as we discussed in Section 5.3, having a user's data in both training and testing can lead to overestimating the model performance.

Many experiments are restricted to controlled lab settings and do not consider anything beyond. This calls for more in-the-wild studies that test the transfer from controlled lab environments to realistic application scenarios. In Sections 3.2 and 4.3.2, we saw that the majority of publications focused on binary tasks. For example, a binary classification of confusion does not take additional emotional or mental states that could have similar attributes which would lead to mislabeling other mental states as confusion.

Finally, Ross et al. (2017) defined the “right for the right reason” principle, suggesting that an ML model has truly learned to generalize when it conforms to the knowledge and expectations of domain experts. This requires using interpretability and explainability methods in the utilized or proposed ML systems, which was discussed in Section 5.4.

##### 5.5.1.4 Reproducibility and replicability

Paleyes et al. (2022) discussed some common challenges that practitioners face when deploying ML solutions, and they noted the shortage of reporting deployment experience in academic literature in general. A step prior to actual deployment would be to test the reproducibility, and reblicability of the proposed models to guarantee that there findings hold true and were not highly specific to their setups.

Gundersen and Kjensmo (2018) defined three levels of reproducibility in relation to ML experiments: *Experiment Reproducibility* refers to achieving the exact same results when using the same ML system with the same data; *Data Reproducibility* refers to achieving nearly identical results when using the same data but with a different ML method, ensuring that the insights derived from the data are consistent regardless of the method used; *Method Reproducibility* refers to achieving similar results or findings when using a different ML method on different data, ensuring that the findings are consistent across various datasets and methods. In addition to reproducibility, there is also replicability. According to the Association for Computing Machinery (ACM),^15^ replicability is the ability of a different group to achieve similar results using similar, but distinct, data and methods that they develop completely independently. A lot of venues encourage researchers to share their code base for transparency and to allow the reproduction and replication of their findings.

However, Samuel et al. (2021) argued that just sharing the code base might not be enough to reproduce reported results due to various reasons, such as incomplete and outdated source codes, insufficient description of model parameters, not reporting the required packages and their version, and unavailable datasets. Semmelrock et al. (2023) also shared similar concerns while giving examples of researchers being unable to reproduce their own results. Semmelrock et al. (2023) attributed this to multiple reasons, such as different data, package versions, hardware setups, and non-determinism of ML models, which is why using fixed random seeds is vital. For example, the code link attached to this publication (Kerkouri et al., 2022) states the code will be made available, and has not been updated ever since.

In addition to sharing data and providing a complete, transparent description of the implementation, it is also important to accurately report and analyze the model's performance. However, some publications either did not report the statistical significance of their results or just compared them to chance levels, which could result in an overestimation of the model's performance. For example, in the field of Brain-Computer Interaction (BCI), most publications compare their results against traditional chance levels, i.e., dividing 100% by the total number of classes, but Combrisson and Jerbi (2015) argued that this could only be achieved by having an infinite number of samples and that chance levels are usually higher than expected. The eye tracking community should engage in similar critical discussions in order to promote common practices in evaluating their results. This will ensure a thorough understanding of the performance of current approaches and identify areas that need further investigation. Furthermore, Paleyes et al. (2022) argued that simply reporting evaluation metrics, e.g., model accuracy, is not sufficient for future deployment and that researchers need to define the requirements of their systems. The requirements ensure that the proposed systems align with the needs and expectations of future users and businesses. Requirements can include performance metrics such as accuracy and F1 scores, metrics to measure model fairness and bias, and any business-specific objectives, such as Key Performance Indicators (KPIs).

#### 5.5.2 Ethical considerations

Passive gaze-based interaction builds on the assumption that by monitoring a user's gaze in the background, we can infer and understand their behavior, cognitive state, and a wide array of sensitive information. This is evident from the learning tasks discussed throughout this paper. However, this could raise multiple ethical, legal, and privacy issues (Gressel et al., 2023). These issues become more apparent when passive gaze-based applications are deployed as part of widely used products outside research. This is why researchers should take particular care of the potential privacy and ethical impact of their work. We recommend following what all of the reviewed user studies stated regarding collecting informed consent forms from their participants, receiving approvals from their respective ethical review boards, and adhering to simple common principles, such as the ethics code published by the American Psychological Association (APA).^16^

Furthermore, the possibility of using webcams to record gaze data,^17^ even at a low quality, allows for the easy construction of a scanpath. This would rapidly accelerate the potential deployment of eye tracking capabilities to consumer laptops, tablets, and smartphones. This means that webcam-based eye tracking could lead to understanding a user's cognitive state, emotional state, product preference, gender, age, and a host of other information. The implications of this are vast and warrant careful consideration. However, discussing the ethical and privacy implications of eye tracking research has gained more interest over the last few years. For example, the most recent iterations of relevant conferences, such as ETRA^18^ and UMAP,^19^ encouraged users to consider the impact of their work on privacy, fairness, and future adoption or misuse of their work. We believe that using ML for passive gaze-based applications can have a lot of potential benefits for users and improve their overall experience when interacting with technologies, but we must consider the potentially harmful impact that could arise from their misuse and discuss how we can mitigate this via appropriate measures.

## 6 Conclusion

We conducted a literature review on machine learning applications in scanpath analysis for passive gaze-based interaction. We reviewed 77 publications spanning a ten-year period from 2012 to 2022. Our goal was to provide an overview of the field and highlight areas that could garner more attention in future research. We categorized publications into five research domains and 11 learning tasks. This highlighted that publications concerning certain domains, such as gaming, sports, and linguistics, and learning tasks, such as usability testing and user modeling, were either missing or underrepresented in using machine learning to analyze scanpaths for passive gaze-based interaction, which offers room for future research efforts. We then presented commonly followed machine learning practices in the order of a machine learning workflow, where we discussed the data curation, the learning data preparation, and the learning process. For data curation, we provided guidelines on how to make informed decisions regarding eye tracker frequency, number of participants, and reporting the user study. In addition, we saw that some publications preferred to use already available datasets, so we reported a list of these 23 datasets. For learning data preparation, we provided insights toward scanpath preprocessing and data splits. We discussed the different scanpath features and different scanpath representation formats. We provided insights toward the different strategies for splitting the data into training, testing, and validation subsets, with cross-validation being preferred as opposed to holdout methods. Finally, for the learning process, we found that traditional machine learning models were preferred over neural networks. SVM was the most used machine learning algorithm across all five research domains, and RNNs were the most popular choice for neural networks. We also provided insights toward making an informed decision on how to evaluate the model performance. Afterwards, we finished our review by focusing on emerging machine learning methods, i.e., SSL, transformer-based models, and XAI, by defining each approach and presenting possible future directions for each concerning scanpath processing.

## Author contributions

AM: Conceptualization, Data curation, Investigation, Methodology, Software, Visualization, Writing – original draft, Writing – review & editing. MB: Conceptualization, Funding acquisition, Methodology, Project administration, Resources, Supervision, Writing – original draft, Writing – review & editing. OB: Validation, Visualization, Writing – review & editing. HA: Validation, Writing – review & editing. DS: Conceptualization, Funding acquisition, Project administration, Resources, Supervision, Writing – review & editing.

## Footnotes

^1^ https://app.dimensions.ai/discover/publication

^2^“Eye Tracking” OR “Eye-Tracking” OR “Eyetracking”

^3^(“Eye Tracking” OR “Eye-Tracking” OR “Eyetracking”) AND (“Deep Learning” OR “Machine Learning” OR “Neural Network*” OR “Decision Tree*” OR “SVM” OR “Support Vector” OR “LSTM” OR “CNN” OR “Random Forest*” OR “KNN” OR “MLP” OR “LDA” OR “Cluster*” OR “Unsupervised Learning” OR “PCA”).

^4^Abbreviation for *Preferred Reporting Items for Systematic Reviews and Meta-Analyses*.

^5^ https://www.scopus.com/

^6^ http://portal.acm.org/guide.cfm

^7^ https://pubmed.ncbi.nlm.nih.gov/

^8^Abbreviation for *A MeaSurement Tool to Assess systematic Reviews*.

^9^ https://www.zotero.org/

^10^The quality metrics were acquired in April 2023.

^11^ https://etra.acm.org/

^12^ https://icmi.acm.org/

^13^ https://www.sciencedirect.com/journal/vision-research

^14^ https://gaming.tobii.com/games/

^15^ https://www.acm.org/publications/policies/artifact-review-and-badging-current

^16^ https://www.apa.org/ethics/code

^17^For example: https://webgazer.cs.brown.edu/.

^18^ https://etra.acm.org/2024/

^19^ https://www.um.org/umap2024/
